# Sub-inhibitory antibiotic treatment selects for enhanced metabolic efficiency

**DOI:** 10.1128/spectrum.03241-23

**Published:** 2024-01-16

**Authors:** Sai Varun Aduru, Karolina Szenkiel, Anika Rahman, Mehrose Ahmad, Maya Fabozzi, Robert P. Smith, Allison J. Lopatkin

**Affiliations:** 1 Department of Chemical Engineering, University of Rochester, Rochester, New York, USA; 2 Department of Biology, Barnard College, New York, New York, USA; 3 Cell Therapy Institute, Kiran Patel College of Allopathic Medicine, Nova Southeastern University, Fort Lauderdale, Florida, USA; 4 Department of Ecology, Evolution, and Environmental Biology, Columbia University, New York, New York, USA; 5 Data Science Institute, Columbia University, New York, New York, USA; 6 Department of Microbiology and Immunology, University of Rochester Medical Center, Rochester, New York, USA; China Agricultural University, Beijing, China

**Keywords:** antibiotic selection, antibiotic resistance, metabolism, bacterial evolution, mathematical modeling

## Abstract

Bacterial growth and metabolic rates are often closely related. However, under antibiotic selection, a paradox in this relationship arises: antibiotic efficacy decreases when bacteria are metabolically dormant, yet antibiotics select for resistant cells that grow fastest during treatment. That is, antibiotic selection counterintuitively favors bacteria with fast growth but slow metabolism. Despite this apparent contradiction, antibiotic resistant cells have historically been characterized primarily in the context of growth, whereas the extent of analogous changes in metabolism is comparatively unknown. Here, we observed that previously evolved antibiotic-resistant strains exhibited a unique relationship between growth and metabolism whereby nutrient utilization became more efficient, regardless of the growth rate. To better understand this unexpected phenomenon, we used a simplified model to simulate bacterial populations adapting to sub-inhibitory antibiotic selection through successive bottlenecking events. Simulations predicted that sub-inhibitory bactericidal antibiotic concentrations could select for enhanced metabolic efficiency, defined based on nutrient utilization: drug-adapted cells are able to achieve the same biomass while utilizing less substrate, even in the absence of treatment. Moreover, simulations predicted that restoring metabolic efficiency would re-sensitize resistant bacteria exhibiting metabolic-dependent resistance; we confirmed this result using adaptive laboratory evolutions of *Escherichia coli* under carbenicillin treatment. Overall, these results indicate that metabolic efficiency is under direct selective pressure during antibiotic treatment and that differences in evolutionary context may determine both the efficacy of different antibiotics and corresponding re-sensitization approaches.

**IMPORTANCE:**

The sustained emergence of antibiotic-resistant pathogens combined with the stalled drug discovery pipelines highlights the critical need to better understand the underlying evolution mechanisms of antibiotic resistance. To this end, bacterial growth and metabolic rates are often closely related, and resistant cells have historically been characterized exclusively in the context of growth. However, under antibiotic selection, antibiotics counterintuitively favor cells with fast growth, and slow metabolism. Through an integrated approach of mathematical modeling and experiments, this study thereby addresses the significant knowledge gap of whether antibiotic selection drives changes in metabolism that complement, and/or act independently, of antibiotic resistance phenotypes.

## INTRODUCTION

Antibiotics bind to key microbial targets central to the bacterial cell replication cycle (1). In doing so, antibiotics can both inhibit bacterial growth (e.g., bacteriostatic) and result in active cell death (e.g., bactericidal) (2). As a result, genetic variants that are able to grow despite the presence of an antibiotic can become enriched (3, 4). Indeed, cells that are able to proliferate at concentrations that are otherwise lethal are empirically classified as resistant. This status is traditionally determined based on an increase in the minimum inhibitory concentration (MIC) of the drug in question and can arise due to a variety of mechanisms. Common (referred to herein as “canonical”) resistance mechanisms generally act by minimizing the ability of an antibiotic to bind to its primary target (e.g., target modification, enzymatic inactivation, or altered transport [5–7]). Despite the apparent specificity of these canonical resistance mechanisms, acquiring and maintaining antibiotic resistance is a complex evolutionary process that extends far beyond the individual drug target itself (8, 9).

In addition to their target-mediated effects, canonical resistance mechanisms can indirectly affect cellular metabolism, often imposing a fitness cost attributed to a metabolic burden (10, 11). For example, acquired amoxicillin resistance has been associated with alterations in core metabolic activity, including an increased glucose consumption rate that returned to baseline as cells adapted to the drug (12). The benefit of a particular resistance-conferring mutation can also be environment-dependent (13). For example, the fitness cost of rifampicin resistance decreases in nutrient-poor conditions (14), suggesting that a metabolic component(s) underlies this phenotype. Finally, mutations in regulatory elements that affect antibiotic target or transport protein expression levels likely have broader global effects on cellular metabolism (15–17) due to non-specific changes in cellular resource allocation. Indeed, mutations in the multiple antibiotic resistance (*mar*) operon can result in the over-expression of efflux pumps and outer membrane porins. Bacteria must compensate for these overexpressed proteins in other aspects of metabolism since their production and utilization is an energy-demanding process (18, 19). In all these cases, bacterial growth rates are often assumed to be a suitable overall proxy for an array of potentially complex and interrelated fitness determinants (20, 21) and are commonly used as a readout of these tradeoffs. For example, while resistance mechanisms are advantageous under antibiotic conditions (i.e., diverse mechanisms often lead to comparable growth rates in the face of selection pressure), in the absence of treatment, antibiotic-resistant mutants exhibit widely varying growth rates due to the broad range of consequences on underlying metabolic processes (10, 22).

In addition to the indirect effects of canonical antibiotic resistance on metabolism, growing evidence demonstrates that bacterial metabolism directly contributes to antibiotic lethality(23–26). Bactericidal antibiotics have been shown to preferentially kill metabolically active cells (27), whereas they exhibit decreased efficacy against those that are metabolically dormant (e.g., persister cells [28, 29]). Intuitively, this suggests that antibiotics impose dual counteracting effects: they simultaneously enrich for clones with the fastest growth, yet lowest metabolic activity, within a population. That is, antibiotics may select for cells with enhanced metabolic efficiency, defined as the amount of energy required per unit biomass formed. This improved efficiency may itself serve as a resistance mechanism, regardless of whether it arises through canonical or non-canonical pathways. Consistent with this intuition, a recent study showed that adapting cells to heightened metabolic states under antibiotic treatment selected for variants with mutations in core metabolic genes. Their over-expression resulted in moderate changes in susceptibility despite no obvious growth defect, a hallmark of increased metabolic efficiency (30). However, whether, and to what extent, antibiotics directly select for changes in metabolism, separate from growth, is largely unknown since common growth-based characterization methods (e.g., fitness costs and MIC) do not separably capture changes in metabolism. Indeed, growth and metabolism are not universally strictly coupled; cells with a highly diverse range of metabolic efficiencies (31, 32) may still exhibit identical growth rates. Clearly, our understanding of metabolic adaptation during antibiotic selection, and its potential therapeutic implications, remains unclear (17).

In this work, we observed that previously characterized *Escherichia coli* strains expressing either canonical or metabolic antibiotic resistance mechanisms consistently displayed increased metabolic efficiency compared to a control strain. Specifically, we focused on nutrient consumption as a proxy for metabolic efficiency since substrate consumption kinetics are widely established both computationally and experimentally, and the tradeoff between consumption rate and growth yield (e.g., metabolic efficiency) has been validated in the literature(33–35). Initial characterization suggested that antibiotics preferentially select for bacteria with enhanced metabolic efficiency, which may directly or indirectly contribute to resistance. To further explore this premise, we used a mathematical framework (36) to investigate the interdependent evolutionary dynamics of growth and metabolism in a microbial population under sub-inhibitory antibiotic selection. Sub-inhibitory concentrations are particularly relevant in this context since resistance evolves in increments often attributed to the stepwise accumulation of mutations (and/or acquisition of resistance genes) selected for under antibiotic treatment (37). In that way, even mutations with moderate effects on resistance levels, e.g. those in genes predominantly involved in core metabolism (henceforth referred to as “metabolic genes”), are important components of the antibiotic resistance landscape, potentially providing strains with competitive advantages over their more sensitive kin (38). Modeling results indicated that, under selection by sub-inhibitory bactericidal drug concentrations, antibiotic-adapted cells were able to grow more efficiently, highlighting that metabolism can act as a selective target independent of both growth and antibiotic mechanism. We validated these results using a 21-day adaptive laboratory evolution of *E. coli* in the presence of carbenicillin. We found that increased metabolic efficiency is responsible for increased resistance phenotypes against multiple bactericidal antibiotics. Moreover, restoring efficiency by modulating environmental nutrient conditions can reverse the resistance phenotype. Overall, these results demonstrate that metabolic selection plays a parallel, contributory role in the evolution of antibiotic resistance, and that targeting metabolic efficiency may serve as a viable strategy to restore antibiotic susceptibility in a clinical setting.

## RESULTS

### Adapted mutants exhibit enhanced metabolic efficiency

In a recent study, we independently adapted *E. coli* to three bactericidal drugs in glucose minimal media under increasingly heightened metabolic states (30). Specifically, bacteria were exposed to lethal drug concentrations under incrementally increasing temperatures, which served as a stimulator of global metabolic activity, over 10 days (30). Evolved populations acquired mutations in both canonical resistance pathways (e.g., drug targets, transporters), as well as core metabolic pathways (e.g., TCA cycle, nucleotide metabolism). We overexpressed these mutant genes and their wild-type counterparts on a medium-copy plasmid to investigate their impact on antibiotic susceptibility. In all cases, strains overexpressing mutant variants of either canonical or metabolic genes exhibited modest MIC increases in response to at least one of the selection drugs used. Moreover, sucAM, a strain that overexpresses a mutant *sucA* gene, exhibited a lower respiration rate with minimal growth defects, initially suggesting this strain had increased metabolic efficiency.

**Fig 1:**
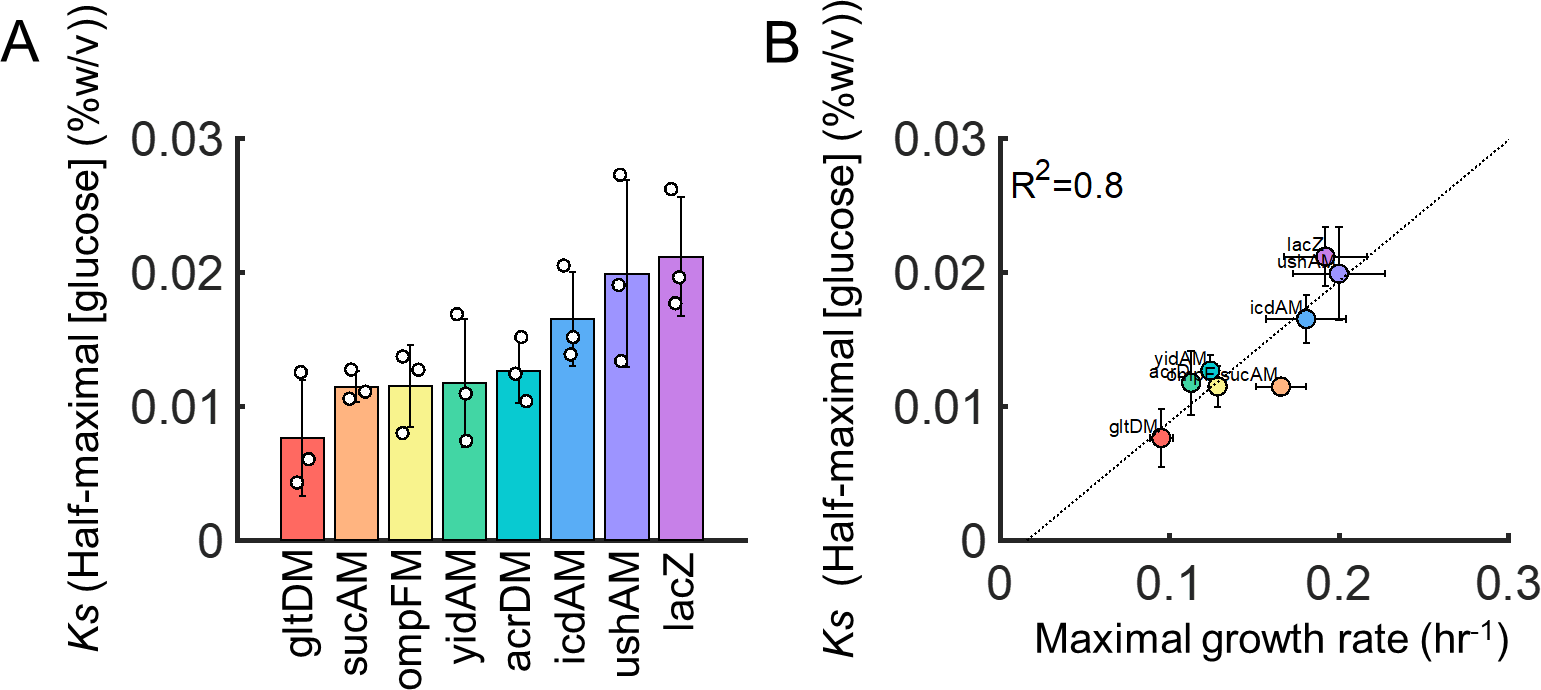
Mutant metabolic efficiency. (**A**) Mutant *K*_S_ values. Half-maximal glucose concentration for all strains calculated based on density at 20 h of growth. For all strains, the *K*_S_ value was statistically equivalent to or less than the control strain (*lacZ*). (**B**) Correlation between *K*_S_ and maximum growth rate. Data from panel **A** plotted as a function of maximum growth rate (i.e., growth in 0.4% glucose). All error bars are standard deviations of three biological replicates.

To further investigate these mutations’ impact on metabolism, here, we quantified each strain’s growth and metabolic phenotype on minimal medium containing glucose as the predominant carbon source (Table S1). We use *K*_S_ as our metric of metabolic efficiency(33–35), defined as the substrate concentration (i.e., glucose) giving rise to half the maximal cell density (or maximal growth rate); practically, *K*_S_ is readily quantified *in vitro*. We found that the control strain expressing the innocuous *lacZ* gene exhibited the greatest *K*_S_ value, while all mutant strains had statistically equivalent (icdAM and ushAM) or lower *K*_S_ values (*P* = 0.0134 one-way ANOVA, Table S2A for individually corrected *P* values) (Fig. 1A). This trend was independent of whether biomass or growth rate was used to calculate *K*_S_ (Fig. S1A), or whether the maximum density was normalized across strains prior to quantification (Fig. S1B). Moreover, while three of the seven strains (icdAM, ushAM, sucAM) showed no statistical difference in growth rate compared to the control, and the other four strains (gltDM, ompFM, acrDM, yidAM) exhibited modest but significant growth defects (*P* = 0.0079, one-way ANOVA, Table S2B for individually corrected *P* values). Interestingly, we observed a statistically significant linear relationship between maximum growth rate (*P* = 0.0029) (or bacterial density after 20 h, *P* = 0.0055) and *K*_S_, suggesting that even for strains with a growth defect, metabolic efficiency improved (Fig. 1B). Together, these results indicated that antibiotics selected for enhanced metabolic efficiency. However, these engineered, overexpressed mutant strains arose in response to lethal antibiotic selection and are not necessarily representative of those that arise following natural selection. Therefore, we next sought to determine whether antibiotics, indeed, select for enhanced metabolic efficiency under these more relevant conditions.

### Model development and characterization

**Fig 2:**
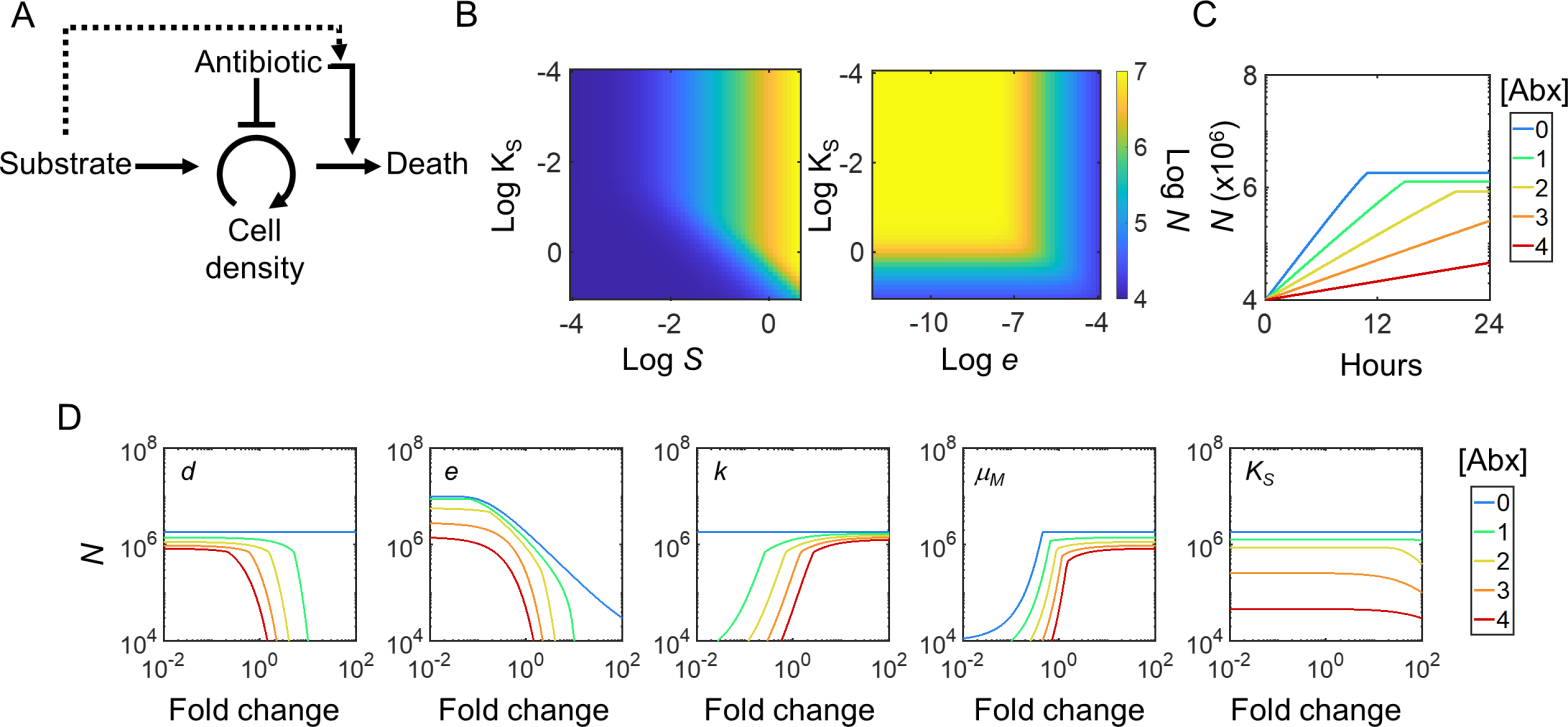
Mathematical model overview and characterization. (**A**) Model network schematic. Substrates are consumed by cells during growth, which results in increased cell density. Antibiotics function by directly inhibiting cellular growth, which reduces cell density, and by promoting cell death. Increasing substrate consumption not only increases growth rate but also makes cells more susceptible to antibiotics. As a result, antibiotics indirectly cause substrate consumption to inhibit growth (dotted lines). (**B**) Metabolic effects in the absence of antibiotics. Initial substrate concentration (*S*) and half-maximal substrate constant (*K*_S_) (left) or metabolic efficiency constant (*e*) (right) are varied 10-fold from their base value. In both panels, log cell density (*N*) is shown after 24 h. (**C**) Antibiotic-mediated growth inhibition. Temporal dynamics of cells in response to sub-inhibitory antibiotic treatment are shown for no antibiotics (*A*), and four representative increasing concentrations (blue, green, yellow, orange, red). (**D**) Parameter intuition under antibiotic treatment. Each of the five main parameters is perturbed ±20% from their base value. Final cell density (*N*) following 24 h of growth in the indicated antibiotic concentration (*A*_Treat_) is shown. Base value of parameters, and simulation conditions, is shown in Table S4.

To investigate how antibiotic selection may affect cellular metabolism, we considered the key interactions between growth, metabolism, and antibiotic-mediated lethality (Fig. 2A). We focused on a generic bactericidal antibiotic and assume that it both inhibits the growth of, and actively kills, cells (36). Critically, increased substrate consumption not only increases growth rate but also makes cells more susceptible to drug-mediated killing—as described above, substrate consumption is a proxy for cellular metabolism, which potentiates lethality (27).

To capture these core interactions, we modified a previously described population-level model consisting of two ordinary differential equations that capture cell density (*N*) and substrate concentration (*S*) over time (36). For this full model, cells (*N*) grow according to classical Monod kinetics, which are described by three parameters: the substrate conversion constant (which describes the fraction of substrate converted into biomass [*e*]), the maximal growth rate (*μ*_M_), and the half-maximal substrate concentration (*K*_S_). We assume that the antibiotic (*A*) both inhibits the growth of, and actively kills, cells according to the IC_50_ concentration (*k*) and specific death rate (*d*), respectively (equations 1-4 (39, 40). However, we implement these mechanisms separately to allow for independent investigation of each: *A*_Eff1_ depends only on *A*_1_, which corresponds strictly to growth inhibition; *A*_Eff2_ depends only on *A*_2_, which corresponds strictly to cell killing. Consistent with literature, we further assume that only bactericidal action directly depends on the metabolic state of the cell (41), independent of growth. Accordingly, we scale the specific death rate *d* by substrate utilization/conversion—greater consumption, corresponding to heightened metabolic states, potentiates antibiotic-mediated cell death. Therefore, the full model corresponds to *A = A*_1_ = *A*_2_ since bactericidal drugs likely impact cells through both growth inhibition and lethality. Finally, *ρ* is a volumetric scalar that ensures modeling results remain consistent with experiments. This model framework is consistent with previous experimental results, which have shown that antibiotics can affect growth (42), metabolism (25), and antibiotic-specific targets that lead to active cell death (5).


(1) $$\frac{dN}{dt}=\left(1-\frac{N}{N_{m}}\right)N\mu_{M}\left(\frac{S}{S+K_{S}}\right)A_{Eff1}-NA_{Eff2}$$

(2) $$\frac{dS}{dt}=-N\mu_{M}\frac{eS}{S+K_{S}}$$

(3) $$A_{Eff1}=\left(\frac{k}{A_{1}+k}\right)$$

(4) $$A_{Eff2}=dA_{2}\left(\frac{eS\rho }{S+K_{S}}\right)$$

Cells grow until all nutrients are exhausted, or space is limited, which is dictated by the initial substrate concentration (*S*_0_) and the carrying capacity of the environment (*N*_m_), respectively. Intuitively, decreasing the half-maximal substrate concentration (*K*_S_) generally increases the final cell density since cells are able to grow faster while consuming lesser amounts of nutrients (43, 44). As expected, this relationship depends directly on the initial substrate concentration, *S*_0_, and inversely on the substrate conversion constant, *e* (Fig. 2B). Further, this relationship is dependent on the antibiotic concentration *A* (Fig. S1C); in the absence of antibiotic treatment, the relationship is independent of the intrinsic death rate (Fig. S1D).

Our model, as expected, predicts that cells exhibit an antibiotic-concentration-dependent decrease in both observed growth rate and maximum density (Fig. 2C). Moreover, the combined interactions between *e*, *μ*_M_, *d*, *k,* and *K*_S_ determine the degree of antibiotic lethality (Fig. 2D). Combined, this model intuitively captures bacterial growth dynamics and resource utilization in the absence and presence of antibiotic treatment using a generic bactericidal drug.

### Simulating adaptive evolution reveals that antibiotics select for enhanced metabolic efficiency

We next explored the interactions between growth and metabolism parameters, and their contribution to the adaptive evolution of antibiotic resistance. During positive selection (e.g., antibiotic exposure), evolution is primarily driven by the combination of mutations with small fitness effects that commonly occur and accumulate over time, along with larger fitness effects that occur less frequently, but can rapidly sweep through a population (45). To account for all these potential scenarios, we modified the model structure to allow for multiple emergent populations growing on the same nutrient source, thereby accounting for both evolutionary (e.g., mutations resulting in adaptive changes over time with the potential for strong selective sweeps) and ecological (e.g., competition and extinction of individual lineages) dynamics.

**Fig 3:**
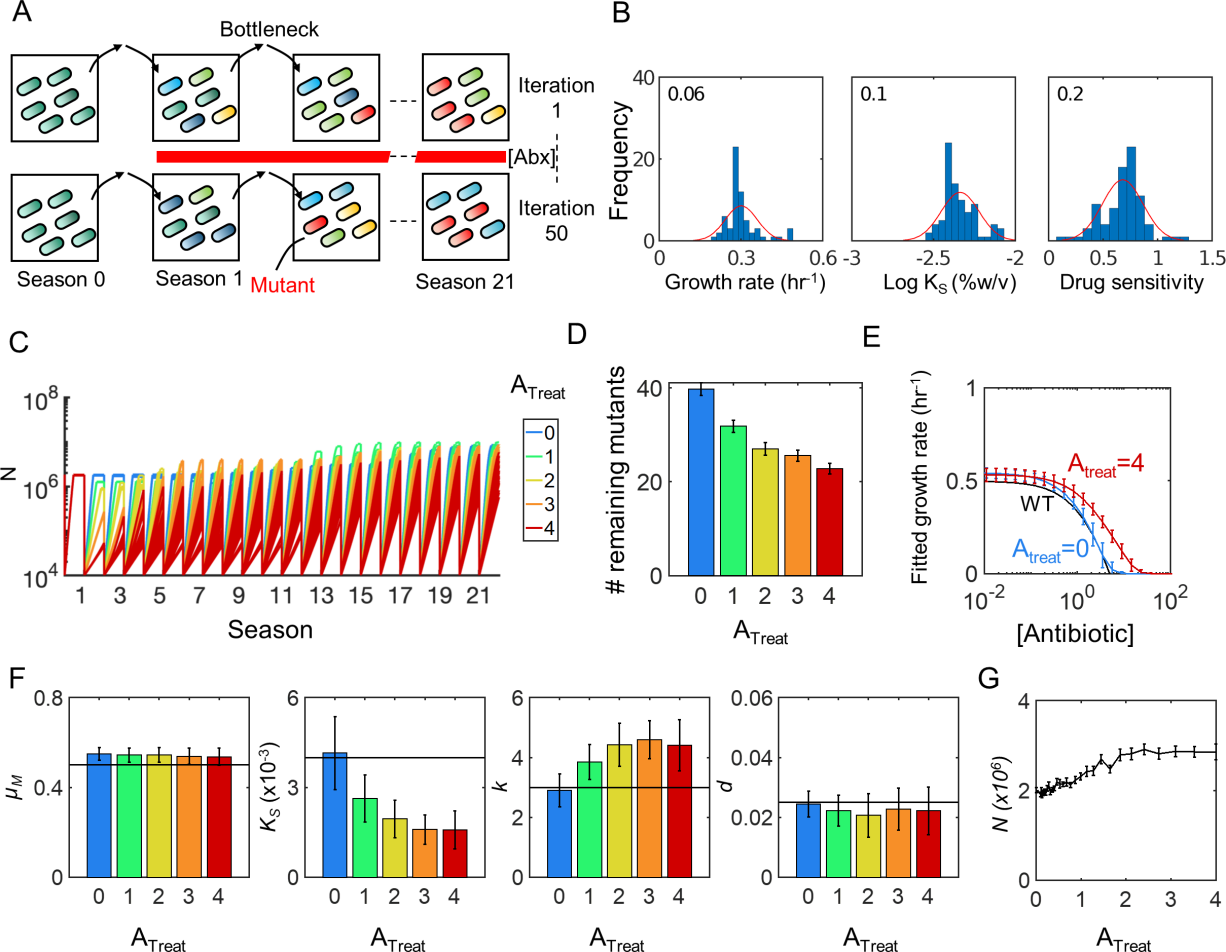
Simulations predict antibiotics select for enhanced metabolic efficiency (**A**) Evolution schematic. A population of cells is pooled and grown for one round in the absence of antibiotics to allow for heterogeneity to emerge (season 0). Following this pre-growth phase, antibiotics are introduced at a constant concentration throughout the duration of the evolution. Fifty iterations are performed for all evolutions to account for variation in the dynamics. (**B**) Parameter distributions. Fifty populations of bacteria are quantified to obtain parameter shapes and variations. Distributions for growth rate *μ*_m_ (left), *K*_S_ (middle), and antibiotic sensitivity as estimated by the fold change in cell density grown at 0 and that grown at 1.6 (right) are shown. (**C**) Temporal evolutionary dynamics. Evolution simulations are performed for five representative, sub-lethal antibiotic concentrations. Cell density dynamics are shown over all 21 seasons. (**D**) Mutant dynamics. A number of mutants that emerged and remained in the population on the last day of the evolution are shown (*y*-axis) at each antibiotic treatment condition (*x*-axis). High concentrations select for fewer number of mutants. (**E**) Evolved IC_50_ concentrations. Populations from the last day of the evolution are used to estimate IC_50_ values. *x*-axis is antibiotic concentration and *y*-axis is growth rate. IC_50_ values are measured for the drug-free (blue) evolution and the treated evolution (*A*_Treat_ = 4) (red). Cells evolved under *A*_Treat_ = 4 exhibit a shifted IC_50_ curve. (**F**) Evolved parameters. On the last day of the evolution, parameter values are collected and weighted by the respective population size. Since substrate utilization is coupled to *K*_S_, only the latter is shown. (**G**) Antibiotics select for increased metabolic efficiency. Metabolic efficiency is defined as the amount of total biomass that is generated when half of the initial substrate is utilized. As antibiotic concentration increases, metabolic efficiency also increases. In all cases, error bars represent standard deviation across all 50 iterations.

We simulated the evolution of a heterogenous population undergoing cyclical rounds of growth (seasons) separated by bottleneck events (Fig. 3A) based on randomized parameters drawn from distributions parameterized by experimental data (Fig. 3B) (see Materials and Methods). Evolutions were simulated over 3 weeks (21 seasons) at five sub-inhibitory antibiotic treatment concentrations (*A*_Treat_) including the antibiotic-free control (*A*_Treat_ = 0). Under all *A*_Treat_, populations reached approximately the same, or greater, density by the last season relative to day 0 (Fig. 3C). Within each population, fewer mutants remained for increasingly higher *A*_Treat_ (Fig. 3D). This is consistent with the known higher likelihood of enriching for rare mutants with increasing selection strength (46). When *A*_Treat_ exceeds the adaptation limit, however, no mutants are selected for and the entire population is eliminated (Fig. S1E). The populations evolved under the highest sub-inhibitory antibiotic concentration (*A*_Treat_ = 4) exhibited modestly shifted dose responses compared to no antibiotic (*A*_Treat_ = 0), confirming the reduction in drug sensitivity (Fig. 3E).

*A*_Treat_-dependent changes in post-evolution parameter distributions revealed the underlying selection targets for these phenotypic shifts (Fig. 3F). In particular, whereas maximum growth rate (*μ*_M_) decreased modestly, *K*_S_ decreased monotonically (*P* < 1e−4), with increasing *A*_Treat_, compared to the untreated control (*A*_Treat_ = 0). Additionally, cells acquired antibiotic resistance via canonical pathways, as confirmed by a significant monotonic increase and decrease in the average IC_50_ (*k*) and death rate (*d*) of evolved populations, respectively (see Table S3 for corrected *P* values). This increase appeared biphasic, and ultimately changes in metabolism were outweighed by canonical resistance (i.e., *k* and *d*) once *A*_Treat_ was sufficiently high.

To confirm that changes in *K*_S_ indicate an increase in metabolic efficiency, we defined various *K*_S_-independent metrics of metabolic efficiency that capture biomass per unit substrate (see Materials and Methods) and investigated the relationships between each metric and *K*_S_ in the absence of evolution. We found that *K*_S_ was strongly inversely correlated with each metabolic efficiency metric, with a Pearson correlation coefficient >−0.97016 in all cases (Fig. S2A through H). Moreover, since *in vitro K*_S_ values inherently reflect the impact of underlying *e*, we expect strains with higher *e* values to evolve towards lower *K*_S_ in a given set of nutrient conditions. Indeed, the two parameters also exhibited a strong inverse correlation (Fig. S2I through L). Finally, to verify that post-evolution reductions in *K*_S_ reflect increased metabolic efficiency of the entire population, we simulated one additional season of each evolved population over a more granular range of *A*_Treat_ and examined one metabolic efficiency metric (i.e., the cell density corresponding to when half the initial substrate was consumed). Results confirmed that increasing *A*_Treat_ results in enhanced metabolic efficiency for the evolved population (Fig. 3G).

Importantly, these results—that metabolic efficiency is selected for under antibiotic selection—were largely robust to the model setup: changes in selection parameters’ mean and standard deviation values (Fig. S3A through H), initial conditions (e.g., lower initial *S*_0_, Fig. S4A through E), and other evolution parameters (e.g., mutation rates, Fig. S4F), did not qualitatively change key results. Although one case (Fig. S3H) suggested metabolic efficiency apparently decreases in the presence of the antibiotic, the parameter evolution (Fig S3I through K) shows that *K*_S_ consistently decreased for all the three cases. Thus, this condition likely captures a regime where our chosen metric may not perfectly capture metabolic efficiency. Additionally, the trend was also lost when the initial substrate is sufficiently low (*S* = 0.01, Fig. S4D). However, under these conditions, growth is severely limited (Fig. S4E), likely preventing complete evolution under the same time period. Moreover, we note that our key results are not dependent on specific model formulations [Fig. S5(i) and (ii), equations 5-8]: two alternative yet consistent frameworks, each capturing diverse representations of metabolic-dependent antibiotic lethality, both predict the same conclusions.

Although both *k* (IC_50_) and the *d* (death rate) evolved in response to increasing *A*_Treat_, neither of these parameters exhibited as strong a dependency on *A*_Treat_ as did *K*_S_. To ensure that multiple putative resistance pathways (*K*_S_, *k*, *d*) were not “competing” with each other and skewing our conclusions, we examined the individual roles of each antibiotic type on these outcomes. While the full model captures a generic bactericidal antibiotic (referred herein as cidal) that may result in both growth inhibition and active cell death, a strict separation of bacteriostatic (referred herein as static) and cidal activity can be achieved by assuming only growth inhibition via the IC_50_ (*k*, static), *or* cell death via the death rate (*d*, cidal). While complete separation of the two may not always be biologically relevant (i.e., actively killing cells likely also inhibits growth), *A*_2_ = 0 and *A*_1_ > 0 corresponds to an entirely bacteriostatic activity, whereas *A*_2_ > 0 and *A*_1_ = 0 corresponds to entirely cidal activity. Intuitively, since only cidal drugs are known to depend on metabolism, we hypothesized that *A*_2_ drives the observed changes in *K*_S_ rather than *A*_1_. Therefore, setting *A*_1_ = 0 should maximize the selection effect on *K*_S_, whereas setting *A*_2_ = 0 should maximize the evolution of the canonical parameter *k*. Indeed, consistent with our interpretation, under entirely static conditions (i.e., *A*_2_ = 0), only *k* evolved significantly, with negligible changes in *K*_S_ (Fig. S6A, *P* = 0.08 and *P* = 5.65e−20 for *K*_S_ and *k*, respectively, one-way ANOVA). In contrast, under entirely cidal conditions (i.e., *A*_1_ = 0), even though *d* decreased modestly, *K*_S_ monotonically and significantly decreased with increasing *A*_Treat_ (Fig. S6B, *P* = 6.55e−28 and *P* = 0.05 for *K*_S_ and *k*, respectively, one-way ANOVA). Overall, these results highlight that cidal drugs specifically select for enhanced metabolic efficiency even in the absence of canonical resistance.

### Experimental evolution confirms modeling predictions

**Fig 4:**
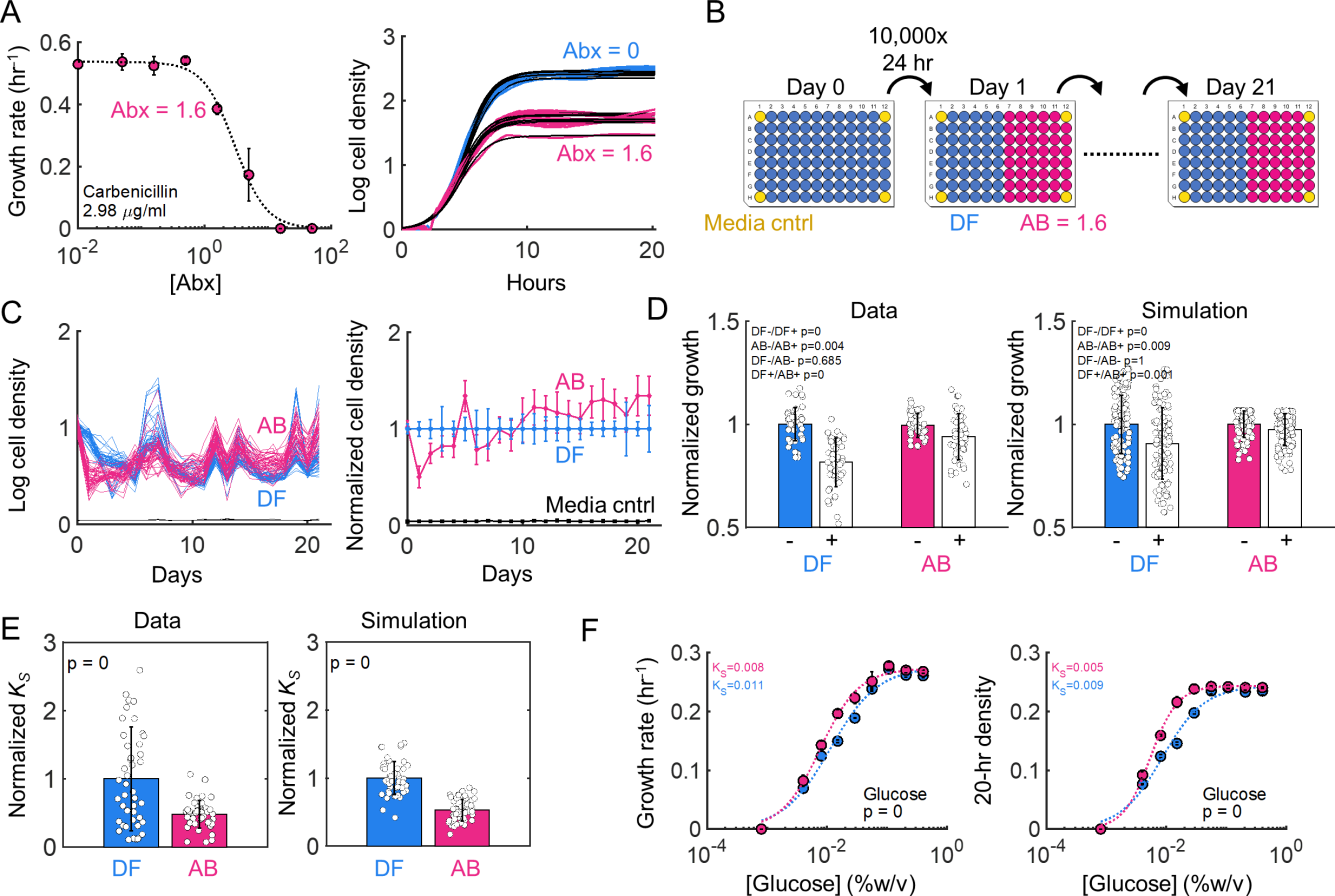
Experiments validate model predictions (**A**) *E. coli* IC_50_. IC_50_ is calculated to determine the appropriate treatment concentration. Concentration of 1.6 µg/mL is chosen for evolution. (**B**) Evolution schematic. A 96 well plate was used to propagate 46 populations of *E. coli* for 21 seasons. Prior to the evolution, cells were grown for one season in the absence of any antibiotic, consistent with modeling, to equilibrate cells to the environment. Four corners of the plate were left cell-free to confirm there was no contamination (cntrl). (**C**) Evolution time course. Cells evolved in 1.6 µg/mL carb are shown in red, and drug-free in blue, over the course of the evolution. Left shows log-transformed raw data and right is normalized to the drug-free population. (**D**) Model predictions and experimental validation for sensitivity. Model predicts that cells moderately adapt to the treating antibiotic, confirmed by experiments. Error bars represent the standard deviation of all 46 populations. (**E**) Model predictions and experimental validation for efficiency. Model predicts that antibiotic treatment selects for lower *K*_S_, implying better growth on lesser amount of carbon, for all 46 populations. Error bars represent the standard deviation of all 46 populations. (**F**) High-resolution *K*_S_ values: Verification of *K*_S_ shift using high-resolution curves and three methods of growth quantification (20-h density, manual growth fit, and logistic growth fit); data confirms 46-population data. Data represent the mean, and error bars the standard deviation, of four biological replicates.

That antibiotics select for cells with increased metabolic efficiency makes intuitive sense: reducing metabolic rates reduces the killing effect of bactericidal drugs, while maximizing growth is favored in competitive environments. However, that this may happen regardless of the canonical mechanism (e.g., any change in *d* or *k*) is surprising. Indeed, simulations revealed that under sub-inhibitory concentrations, there were no, or weak, correlations between metabolic and resistance parameters within a population (e.g., *K*_S_ and *k*, or *K*_S_ and *d*, *R*^2^ ≤ 0.2 in all cases) (Fig. S6C and S6D). This suggests that antibiotics select for enhanced efficiency as a general phenotype, in parallel with, and agnostic to, any resistance that evolves through diverse canonical mechanisms. Thus, we next sought to investigate whether *in vitro* systems, subject to natural biological constraints, would confirm our modeling conclusions. Since our model equations are not specific to any particular cidal mechanism of action, we chose the antibiotic carbenicillin, which belongs to one of the most widely used antibiotic classes and also has been shown to exhibit clear metabolic-dependent efficacy (30, 47). To select an appropriate sub-inhibitory concentration, we quantified the IC_50_ of the *E. coli* strain BW25113, from which a sub-inhibitory concentration of 1.6 µg/mL was chosen (Fig. 4A).

We designed and implemented an *in vitro* evolution consistent with the model setup (Fig. 4B). Specifically, an isogenic population of BW25113 was first propagated for one season in glucose minimal media (referred to as day 0) in the absence of antibiotics in a 96 well plate, where 46 replicate populations were assigned to each of two treatment groups (drug-free and abx denote carbenicillin = 0 and 1.6 µg/mL, respectively). The four corners of the plate were left cell-free and served as a contamination control. This 24-h population’s parameter distributions were used to initialize the model (i.e., Fig. 3B corresponds to day 0 measurements); the population was used to initiate longitudinal evolution. Following the first day (i.e., one 24-h “season”) of growth in the absence of any drug, cells were diluted 10,000-fold into fresh glucose minimal media, and either sterile water or drug was added to half of the plate. This dilution was repeated daily for a total duration of 21 days (equivalent to 21 seasons in the model). All populations were sampled and stored in glycerol on day 0 and day 21 for subsequent characterization. We refer to populations that evolved in the absence of drug as DF (drug-free) and those that evolved in the presence of carbenicillin as AB (abx-treated), for consistency.

*In vitro* evolutionary dynamics were consistent with model results, where, only over a relatively short initial timeframe did AB populations exhibit a decrease in density relative to DF (Fig. 4C). Ultimately, the relative density of the AB group exceeded that of DF (*P* = 6.64e−100, two-tailed *t*-test), initially suggesting improved metabolic efficiency under antibiotic selection. Specifically, we examined the growth rates of populations evolved under DF or AB condition, in the absence/presence of antibiotic (±) (Fig. 4D); we note that this captures drug sensitivity as in Fig. 4A (right). Both DF and AB populations exhibited statistically equivalent growth rates in the absence of the drug, consistent with modeling results that predicted minimal change in drug-free growth regardless of evolution conditions. Similarly, AB populations grew moderately faster under 1.6 µg/mL carbenicillin as compared to their DF counterparts, confirming that AB populations evolved moderate resistance under these conditions.

Next, we compared changes in metabolism for all 92 populations. We used a coarse range of glucose concentrations to estimate changes in *K*_S_ (5 concentrations including 0 glucose as control) for all 92 populations, along with the wild type (WT). Indeed, both the model predictions and experiments confirmed that, on average, the 46 AB populations exhibited a statistically lower *K*_S_ compared to the 46 DF populations (Fig. 4E), as quantified from equation 6 (Materials and Methods). These results were not an artifact of the glucose concentrations chosen. Indeed, randomly selecting and characterizing the *K*_S_ of 6 populations with higher glucose resolution confirmed that AB populations exhibited a significantly reduced *K*_S_ compared to DF. This was true regardless of whether *K*_S_ was measured based on growth rate (Fig. 4F, left) or cell density (Fig. 4F, right). Overall, these results are consistent with initial mutant characterization (Fig. 1), and confirm that, in parallel with growth-based selection, sub-inhibitory carbenicillin selects for cells with increased metabolic efficiency.

### Restoring metabolic efficiency reverses antibiotic resistance phenotypes

**Fig 5:**
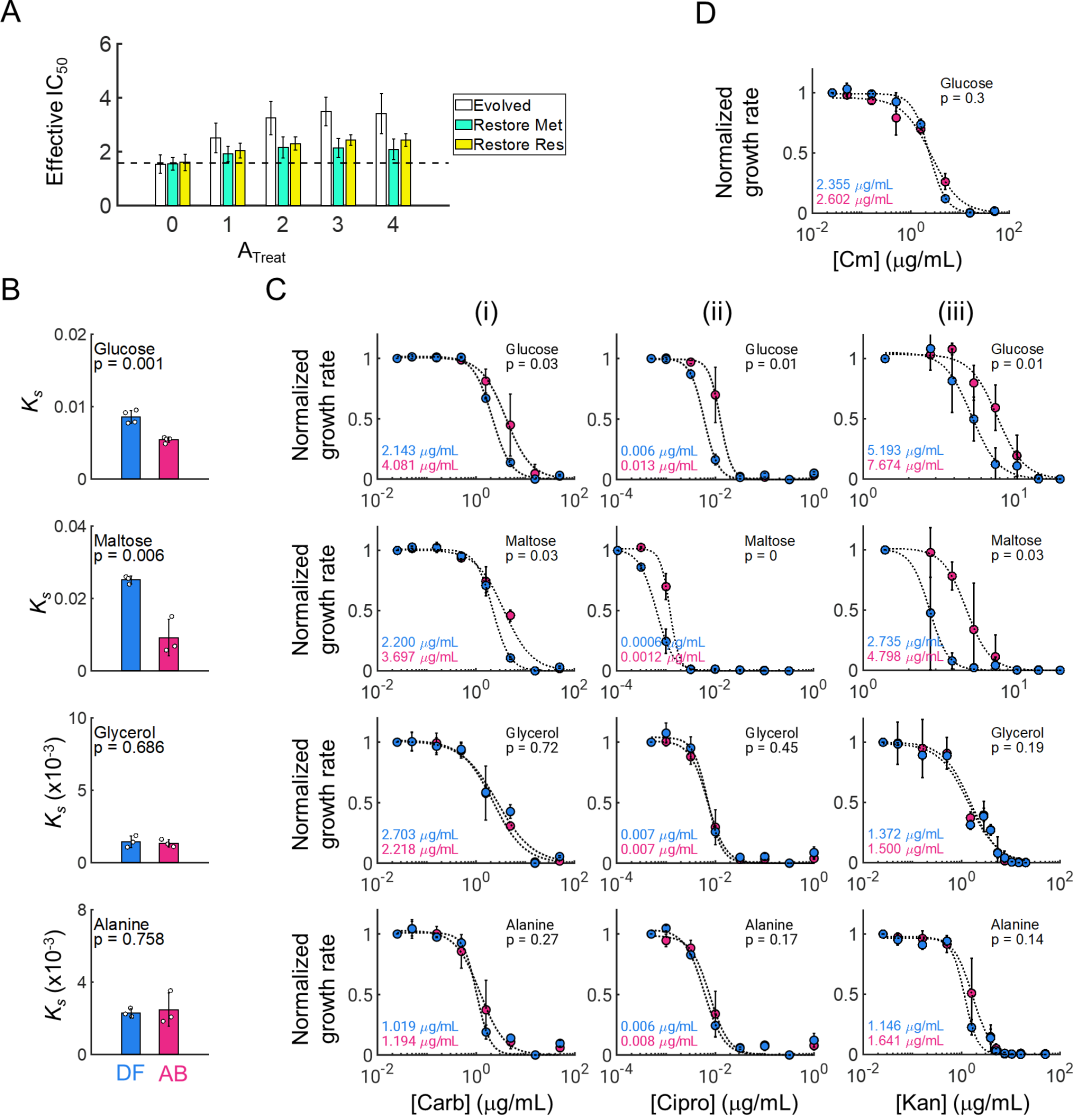
Re-sensitizing cells using alternative carbon sources. (A) Re-sensitization model prediction. Effective IC_50_ values are measured for evolved populations with restored metabolism parameters (*K*_S_ and e; green), restored resistant parameters (k and d; yellow), or no change (white). P values are shown for lsd-corrected two-sided t-tests. (B) Restoring sensitivity by changing *K*_S_. *K*_S_ values for drug-free (DF) and antibiotic-treated (AB) populations are measured in glucose, maltose, glycerol, and alanine (top to bottom). Significant reductions in *K*_S_ are observed in glucose and maltose only. Bar heights represent the means of three independent replicates (i.e., three unique populations), and white markers indicate individual values. (C) IC_50_ values for the same three DF and AB populations (blue and pink, respectively) are measured for carbenicillin (i), ciprofloxacin (ii), and kanamycin (iii). Carbon sources used are shown in the top right of each plot, and P values are shown for Bonferroni-corrected two-sided t-tests. (D) IC_50_ for bacteriostatic antibiotic. IC_50_ in glucose of chloramphenicol for the same three populations. For panels B and C, markers and error bars represent the mean and standard deviation of three biological replicates.

Based on these results, it is clear that *K*_S_ evolves as a result of antibiotic selection. However, whether changes in *K*_S_ alone are responsible for conferring phenotypic resistance, or are merely byproducts of canonical resistance acquisition, is not yet clear. We next attempted to indirectly separate these possibilities by examining whether restoring baseline metabolic efficiency (i.e., *K*_S_) is sufficient to recapture an antibiotic susceptible phenotype regardless of changes in IC_50_ (i.e., without antibiotic-mechanism-specific intervention). To test this, we tested three scenarios *in silico* using parameter distributions corresponding to day 21 AB populations. Specifically, we quantified the effective IC_50_ as a measure of resistance given the populations had (i) restored metabolic efficiency only (revert only *K*_S_ to ancestral values), (ii) restored resistance only (revert only evolved *k* and *d* to ancestral values), or (iii) nothing (i.e., maintain same parameter values as on day 21). Results confirmed that, for all values of *A*_Treat_, restoring drug susceptibility could be achieved by re-sensitization through either metabolic or canonical resistance pathways (Fig. 5). For a population evolved under a strictly cidal drug (*A*_1_ = 0), restoring metabolic parameters was sufficient to entirely re-sensitize the population, whereas restoring canonical resistance parameters had minimal effect (and vice versa for static drugs) (Fig. S6E and S6F). Thus, these results suggest that some re-sensitization strategies depend on the general type of antibiotic to which resistance has been evolved, as well as the environment under which the evolution occurred.

To validate these predictions, we first quantified the IC_50_ of evolved DF and AB populations under identical media conditions as in the evolution (i.e., minimal media with glucose as the primary carbon source). In this environment, as expected, AB populations exhibited optimized metabolic efficiency (i.e., reduced *K*_S_) (Fig. 5, glucose), and resistance to the bactericidal antibiotic carbenicillin (i.e., increased IC_50_) (Fig. 5, top row), relative to the DF population. Importantly, however, this finding was not specific to carbenicillin, and the same AB populations exhibited increased IC_50_ values to both ciprofloxacin and kanamycin, which represents the three main bactericidal antibiotic classes (Fig. 5, top row). Next, to confirm these results were not an artifact of the glucose-specific evolution conditions, we looked for alternative carbon sources that would maintain AB’s lower *K*_S_ relative to DF. Indeed, the glycolytic carbon source maltose, which enters glycolysis immediately following glucose (48), also resulted in reduced *K*_S_ for AB compared to DF populations (Fig. 5, second row). Moreover, as with glucose, the IC_50_ of AB was statistically greater than DF for all three antibiotics, consistent with our hypothesis (Fig. 5, second row). In contrast, both glycerol and alanine resulted in no discernible *K*_S_ differences between AB and DF (Fig. 5, bottom two rows). Consistent with our interpretation, these populations also had statistically indistinguishable IC_50_s under all three drugs, indicating that susceptibility was fully restored in these environments (Fig. 5, bottom two rows). Overall, only carbon sources that restored *K*_S_ to the DF value led to restored IC_50_ values.

Finally, to confirm that the restoration of antibiotic susceptibility was a result of metabolic-specific shifts in efficiency driven by metabolic-dependent (cidal) antibiotics, we tested whether these trends would be maintained by drugs that do not affect bacterial metabolism (static). Specifically, we used chloramphenicol, a bacteriostatic antibiotic whose sensitivity should not depend on the local *K*_S_ value and measured IC_50_s in glucose media. Results confirmed that, while AB and DF have distinct *K*_S_ values in this environment, there was no detectable shift in IC_50_ (Fig. 5). Overall, these confirm that metabolic-dependent antibiotics can induce selection dynamics based on altering underlying metabolic physiology, complementary to resistance evolved to specific drug mechanisms.

## DISCUSSION

Antibiotics are simple small molecules, yet their effects on bacterial populations are highly complex and often unpredictable. Understanding various drugs’ underlying physiological consequences, resulting population dynamics, and evolutionary outcomes, can, provide insights into methods that leverage microbial behavior into more efficacious treatments strategies. Such strategies, including the development of alternative dosing regimens (49), combination treatments (50), rational antibiotic stewardship approaches (51), and others that may significantly contribute to blunting the threat of antibiotic resistance, have garnered recent interest. To date, such advances have been the result of a primary research focus on canonical, mechanism-based antibiotic selection and resistance. However, despite growing evidence of their importance, the effects of selection on metabolic traits remain largely unexplored.

Here, to better understand the potential role of general metabolism as a target of antibiotic selection, we developed a modeling framework that captures ecological responses to a generic bactericidal antibiotic, along with its selection-dependent evolution. Critically, we emphasize selection dynamics due to sub-inhibitory drug concentrations, which are inherently more likely to lead to the types of metabolic adaptations that may be readily overlooked/shadowed by classical approaches. Specifically, modeling results indicate that metabolic efficiency, described by the nutrient half-maximal growth constant *K*_S_, increases (since *K*_S_ decreases) as a consequence of selection by sub-inhibitory bactericidal antibiotic conditions. This decrease in *K*_S_ corresponds to an enhanced observed IC_50_ despite negligible changes in drug killing and inhibition parameters. We confirmed these predictions *in vitro*, using the bactericidal drug, carbenicillin. A statistically significant decrease in *K*_S_ following a 21-day adaptation to sub-inhibitory carbenicillin corresponded to a significantly shifted susceptibility profile.

Interestingly, according to the modeling results, metabolic efficiency was positively selected under bactericidal, not bacteriostatic, drug selection. This makes intuitive sense, given that bactericidal antibiotic lethality has been shown to depend directly on the cell’s downstream metabolic response to drug, which does not occur in response to bacteriostatic antibiotics (23, 52). That model-predicted resistance did not arise primarily due to drug-target-specific mutations/adaptations suggested that susceptibility could be restored under environmental conditions corresponding to a reversion to baseline efficiency. Indeed, we showed this to be the case; carbenicillin resistance was abolished in nutrient conditions mimicking original *K*_S_ values, and this reversal was maintained for additional bactericidal antibiotics. Moreover, dose responses in glucose to the bacteriostatic antibiotic chloramphenicol were unchanged between DF and AB populations. Together, these results suggest that compounds which manipulate metabolic efficiency, rather than growth, represent an exciting potential path to increasing, restoring, or maintaining antibiotic susceptibility. Indeed, strategies to re-sensitize bacteria, including metabolites and small molecules, are being implemented as antibiotic adjuvants to improve antibiotic efficacy (53, 54). However, as demonstrated here, by focusing on modulating complex metabolic phenotypes, rather than metabolite-specific network pathways, adjuvants may eventually be more broadly applicable to entire classes of drugs, making them a key direction of further mechanistic and translational research.

A thorough sensitivity analysis allowed us to interpret the robustness of these results. Overall, the results were qualitatively maintained across a broad range of parameter values and model formulations. However, there are some limitations. First, the evolutionary setup used a simplified representation of natural evolution. The model assumes only one mutant per sub-population emerges per season, which is not necessarily representative of natural bacterial evolution that typically involves a greater degree of heterogeneity. Second, select parameter regimes did result in qualitatively different outcomes. For instance, Fig. S3H through I illustrate a scenario where a decrease in *K*_S_ does not necessarily correspond to an increase in metabolic efficiency, pointing out a limitation in using *K*_S_ as a proxy metric. Similarly, there’s no marked rise in the metabolic efficiency while exploring the full parameter space of *k/*IC_50_. Finally, we note the model assumes generic action of an antibiotic and highly simplified definitions of “metabolism” (i.e., that it is defined by substrate uptake only). Incorporating more mechanistic detail of specific drug types and corresponding metabolic dependencies may reveal more insights into the ways in which the parameters evolve.

Overall, this study emphasizes the need to consider the metabolic implications of microbial evolution in the face of antibiotic treatment. We have shown that cellular metabolism is a direct selection target, both in an idealized setting and in a more biologically feasible framework and demonstrated that antibiotic selection results in selection for enhanced metabolic efficiency overall. We note that myriad other factors (e.g., spatially imposed heterogeneity and fluctuating environmental nutrients/stressors) undoubtedly contribute to metabolic adaptation over time. Thoroughly characterizing the evolutionary relationship between metabolism and growth in bacterial populations under antibiotic selection, both experimentally and with increasing modeling complexity, is, therefore, a critical next step in developing effective antibiotic treatments.

## MATERIALS AND METHODS

### Evolutionary model

Each simulation was initialized assuming a clonally homogenous population of 10^4^ unique cells with normally distributed phenotypic heterogeneity. Parameter values for each cell were drawn from distributions approximated from experimental data according to their calculated means and standard deviations (Fig. 3B). Specifically, we split one clone of the *E. coli* strain BW25113 into 92 distinct replicates and measured the expected variance in growth, metabolism, and antibiotic sensitivity for each corresponding to the day 0 plate. These standard deviations were used to set widths, and average values were used to set centers, of each base parameter distribution. Values for *μ*_M_, *K*_S_, and *k* were determined directly from the data by quantifying maximum growth rate, half-maximal concentration of glucose utilization, or sensitivity to 0.5× the IC_50_ of carbenicillin, respectively. Since *k* and *K*_S_ each represent drug sensitivity and metabolism variation, *d* and *e* could then be approximated using the same variance, with means shifted based on literature values. We note that this assumption of equal variances does not qualitatively change the overall metabolic efficiency profiles (Fig. S3A and S3C). Next, we conservatively assume that *de novo* mutants arise at a frequency of 0.003 per gene per cell (55, 56). To calculate the number of mutants present at the end of each season, we calculate the number of generations that occurred and generate a binomial distribution for every unique cell given a probability equal to the generation number multiplied by mutation rate. Any cell that is assigned a value of 1 is then used to draw new parameters for its corresponding mutant from the relevant distribution at the time the mutation arose. If the antibiotic concentration proves lethal thereby eliminating all the mutants, we assign the number of mutants as zero. Bottleneck events are implemented once every 24 h by randomly choosing cells based on the weighted population densities from the end of the previous season. If the cell density fails to plateau at the set season1 cutoff (10^5^) for any season, the respective simulation for the season within the iteration is repeated until the cutoff condition is met. Selected cells (corresponding to individual parameter sets) are then diluted to a preset density to initiate the next season. In all cases, simulations were run for 50 independent iterations to account for variation in parameter selection, and results were averaged across all iterations. For any given season within an iteration, a zero efficiency is assigned if the density does not exist at 50% of the initial substrate concentration. While averaging, all such zero efficiencies were censored. Overall, changes in the average parameters at the end of the evolution reflect both ecology (competition/extinction) and evolution (mutant acquisition) on a clonal starting population. We note that sensitivity analyses confirm that base parameter choices did not impact the main qualitative trends (Fig. S3A through D).

### Definitions of metabolic efficiency

We compared the amount of biomass formed per unit substrate consumed, i.e., the metabolic efficiency, using four metrics (Fig. S2A through H):

1. *N*/*S* at a given timestamp: the ratio of cell density (normalized with respect to its initial value) to substrate concentration was used to denote metabolic efficiency, at any given timestamp.
2. Log *N*/*S* at a given timestamp: the ratio of a natural log transformed cell density (normalized with respect to its initial value) to substrate was used in this case to represent metabolic efficiency. Both for definitions 1 and 2, the substrate *S*, was allowed to evolve with time.
3. *N* with constant and excess *S*: this definition considers metabolic efficiency as cell density when substrate *S* was held constant in excess of 0.5.
4. *N* at specific *S*: the cell density *N*, selected at a value of the substrate *S*, represents the metabolic efficiency. Here, we consider the cell density achieved when the substrate was reduced by half of its initial value.

### Alternative model structures

Two additional model variations were considered.

Alternative model #1: here, we assume that bactericidal-mediated cell death is directly proportional to growth inhibition.


(5) $$\frac{dN}{dt}=\left(1-\frac{N}{N_{m}}\right)N\mu_{M}\left(\frac{S}{S+K_{S}}\right)\left(\frac{k}{A_{1}+k}\right)\left(1-d\right)A_{2}e\rho$$

(6) $$\frac{dS}{dt}=-N\mu_{M}\frac{eS}{S+K_{S}}$$

Alternative model #2: here, we assume that bactericidal-mediated death is directly proportional to substrate concentration and conversion but independent of *K*_S_ directly.


(7) $$\frac{dN}{dt}=\left(1-\frac{N}{N_{m}}\right)N\mu_{M}\left(\frac{S}{S+K_{S}}\right)\left(\frac{k}{A_{1}+k}\right)-{dNSA}_{2}e\rho$$

(8) $$\frac{dS}{dt}=-N\mu_{M}\frac{eS}{S+K_{S}}$$

### Long-term evolution

A long-term evolution experiment was carried out using the *E. coli* strain BW25113. Briefly, one clone was picked from a newly streaked agar plate and grown overnight in 2 mL Luria broth (LB) media for 16 h at 37°C with agitation at 250 rpm. The overnight culture was first re-suspended 1:1 (vol/vol) in M9CAG media (M9CA medium broth powder from Amresco, cat # J864-100G, containing 2 mg/mL casamino acid, supplemented with 2 mM MgSO_4_, 0.1 mM CaCl_2_, and 0.4%[wt/vol] glucose), diluted 10,000× in M9CAG media, and 200 µL was distributed into 92 wells of a 96-well plate. The four corners were filled with 200 µL of media only as contamination controls. The plate was then sealed with a Breath-easy sealing membrane (Sigma Aldrich, cat# Z380059) and grown overnight for 23 h at 37°C with agitation at 250 rpm. After 23 h, the plate was removed from the incubator and the optical density (600 nm) was recorded using a Spark Multimode Reader (Tecan, Mannendorf, Switzerland). This plate constituted day 0 (day 0) for subsequent long-term evolution, or for model characterization. To initiate the long-term evolution, samples from the day 0 plate were transferred to a new plate such that all wells were split into two groups corresponding to either drug-free (DF) and antibiotic-treated (AB). Either water (for DF) or 1.58 µg/mL carbenicillin (for AB) populations, respectively, was added to the corresponding wells according to the map in Fig. 3B. Columns 1–6 remained DF and 7–12 corresponded to AB populations. Cells were then transferred using a sterile 96-well replicator. The plate was then sealed and grown for 23 h in identical conditions as described above. The same protocol was followed daily, including optical density readings, for the duration of the experiment. In all cases, the negative control wells were first checked for contamination. In the case where contamination was visible, the most recent bottlenecking event was implemented using the stored plated corresponding to the previous day. In general, on days 0, 4, 7, 11, 14, and 21 of the evolution, the populations were stored by transferring 100 µL from each well to a separate microplate containing 100 µL of 50% (vol/vol) glycerol. After mixing, the plate was sealed with aluminum foil seals (AlumaSeal II, cat# AF100) and stored at −80°C for future use.

### Full-plate (low-resolution) population characterization

To characterize populations on plates day 0 and day 21, up to 8 populations were measured simultaneously on a single 96 well plate. Specifically, each population was inoculated from the stored glycerol plate into 2 mL of LB media using sterile p10 pipette tips. A negative control of 1 mL of LB media was also included to assess for contamination. If growth was observed in the control, the experiment was discarded. Cultures were grown at 37°C for 16 h with 250 rpm agitation. Then, 500 µL of each overnight culture was centrifuged at 10,000 rpm and resuspended in M9 blank media (M9 with no casamino acids or carbon source). Resuspended cells were diluted 1,000× into either M9 blank media for a low-resolution *K*_S_ curve, or M9CAG for maximum growth rate and carbenicillin sensitivity measurements. In both cases, 198 µL of these solutions was aliquoted into each well of a sterile 96 well plates. Columns filled with M9CAG were mixed with either sterile water or 1.58 µg/mL carbenicillin. The columns filled with M9 blank medium were mixed with glucose to achieve the final concentrations of 0.4%, 0.4%, 0.1%, and 0.004% (wt/vol). All growth/metabolism parameters were measured in technical duplicates. Wells were then covered with 50 µL of mineral oil and placed in a temperature-controlled Tecan plate reader where optical density was measured every 15 min for up to 24 h.

### High-resolution *K*_S_ and IC_50_ quantification

For all high-resolution measurements, three populations from DF and AB each were chosen randomly for characterization, where these populations were treated as biological replicates. Specifically, six overnight cultures were set up by inoculating bacteria from wells D2, F3, and C4 for DF and B9, F8, and E11 for AB from the stored day 21 plate into 2 mL of LB media. A negative control of 1 mL of LB media and a bacteria-free sterile pipette tip were included. Cultures were grown at 37°C for 16 h with 250 rpm agitation. Then, 500 µL of each overnight culture was centrifuged at 10,000 rpm and resuspended in M9 blank media (M9 with no casamino acids or carbon source). Resuspended cells were diluted 1,000× into M9 blank media and 198 µL was aliquoted into every well of a 96 well plate. Then, 2 µL of pre-mixed glucose, maltose, glycerol, and alanine was added to each well to achieve the final concentrations of 0.4%, 0.207%, 0.107%, 0.056%, 0.029%, 0.015%, 0.008%, and 0.004% (wt/vol) for glucose and maltose; 0.207%, 0.107%, 0.056%, 0.029%, 0.015%, 0.008%, 0.004%, and 0.002% (wt/vol) for alanine; and 0.107%, 0.056%, 0.029%, 0.015%, 0.008%, 0.004%, 0.002%, and 0.001% (wt/vol) for glycerol. IC_50_ values were obtained using an identical setup, except M9CA-carbon media was used, where carbon consisted of 0.4% of glucose, maltose, alanine, or glycerol. 2 µL of each antibiotic was then added to each well at the following concentrations: 50, 15.8, 5, 1.58, 0.5, 0.16, 0.05, 0 µg/mL for carbenicillin and chloramphenicol; 20, 14.4, 10.4, 7.46, 5.37, 3.86, 2.78, and 0 µg/mL for kanamycin; and 1, 0.32, 0.1, 0.032, 0.01, 0.0032, 0.001, and 0 µg/mL for ciprofloxacin. In both cases, concentrations were chosen to appropriately span dose responses at approximately the center of chosen range. Every population was measured in technical duplicate such that all 6 populations could be measured on the same 96 well plate. All experiments were repeated at least twice to ensure reproducibility.

Once growth curves were complete, technical duplicates were averaged. To quantify growth rate, curves were fitted using the modified logistic growth equation:


(9) $$N=\frac{A}{1+\left(e^{\frac{4\mu_{m}}{A}\left(\lambda -t\right)+2}\right)}$$

where $\mu_{m}$ and λ are taken to be the maximum growth rate and lag time, respectively, *N* is the log-transformed cell density, and *A* is the maximum density achieved. To determine *K*_S_, calculated $\mu_{m}$ was then fit to carbon concentrations using the following equation:


(10) $$\mu_{m}=\frac{\gamma {\left[C\right]}^{n}}{K_{S}^{n}+{\left[C\right]}^{n}}$$

where γ is the maximum growth rate in excess carbon concentrations [*C*], *n* is the hill coefficient, and *K*_S_ is the concentration that corresponds to half the maximum growth rate. Alternatively, to calculate IC_50_, the following equation was used:


(11) $$\mu_{m}=\frac{\gamma {IC}_{50}^{n}}{{IC}_{50}+{\left[A\right]}^{n}}$$

where [*A*] corresponds to the antibiotic concentration. To determine *K*_S_ or IC_50_-based density, the same procedure was used except data were fit to *N*_20_ instead of $\mu_{m}$ , corresponding to the log-transformed cell density at 20 h of growth in the Tecan. Each population was fit individually and averaged together to obtain the mean per treatment group.

## Data Availability

All data generated in this study are provided in the supplemental material. All code for this paper can be found on the lab’s GitHub.

## SUPPLEMENTAL MATERIAL

Supplemental material spectrum.03241-23-s0001.pdf Additional figures and tables for support.

ASM does not own the copyrights to Supplemental Material that may be linked to, or accessed through, an article. The authors have granted ASM a non-exclusive, world-wide license to publish the Supplemental Material files. Please contact the corresponding author directly for reuse.
