# Supplementary material for: Sub-inhibitory antibiotic treatment selects for enhanced metabolic efficiency
Source: Microbiol Spectr. 2024 Jan 16;12(2):e03241-23. doi: 10.1128/spectrum.03241-23 (PMC10846238; doi:10.1128/spectrum.03241-23)
Supplement: Supplemental material — Additional figures and tables for support. [file spectrum.03241-23-s0001.pdf]

Supplementary Information For:

Sub-inhibitory antibiotic treatment selects for enhanced metabolic efficiency

Sai Varun Aduru[1], Karolina Szenkiel[2], Anika Rahman[2], Mehrose Ahmad[2], Maya Fabozzi[2], Robert P. Smith[3], Allison J. Lopatkin[1,2,4-6,*]

[1] Department of Chemical Engineering, University of Rochester; Rochester, NY 14627; USA
[2]Department of Biology, Barnard College; New York, NY 10027; USA
[3]Cell Therapy Institute, Kiran Patel College of Allopathic Medicine, Nova Southeastern University, Fort Lauderdale FL, 33314
[4]Department Ecology, Evolution, and Environmental Biology, Columbia University; New York, NY 10027; USA
[5]Data Science Institute, Columbia University; New York, NY 10027; USA
[6]Department of Microbiology and Immunology, University of Rochester Medical Center; Rochester, NY 14627; USA

[*]Corresponding author: allison.lopatkin@rochester.edu

Table S1: Strains and plasmids used in this study

A. Strains

Designation	Description	Strain Genotype
Ctrl	WT *E. coli* strain BW25113 with pAB191	F^-, □ *(araD-araB)567,* □ *lacZ4787(::*rrnB-3*),* □ $^-$ *, rph-1,* □ *(rhaD-rhaB)568, hsd*R*514,* pAB191
icdAM	*E. coli* strain BW25113 with *icd* knocked out, kanamycin resistance removed and pAB01a	F^-, □ *icd,* □ *(araD-araB)567,* □ *lacZ4787(::*rrnB-3*),* □ $^-$, □ *(icd724), rph-1,* □ *(rhaD-rhaB)568, hsdR514,* pAB01a
ushAM	*E. coli* strain BW25113 with *ushA* knocked out, kanamycin resistance removed and pAB04a	F^-, □ *ushA,* □ *(araD-araB)567,* □ *lacZ4787(::*rrnB-3*),* □ $^-$, □ *(ushA763), rph-1,* □ *(rhaD-rhaB)568, hsd*R*514,* pAB04a, pAB02a
ompFM	*E. coli* strain BW25113 with *ompF* knocked out, kanamycin resistance removed and pAB09a	F^-, □ *ompF,* □ *(araD-araB)567,* □ *lacZ4787(::*rrnB-3*),* □ $^-$, □ *(ompF746), rph-1,* □ *(rhaD-rhaB)568, hsd*R*514,* pAB09a
sucAM	*E. coli* strain BW25113 with *sucA* knocked out, kanamycin resistance removed, andpAB02a	F^-, □ *sucA,* □ *(araD-araB)567,* □ *lacZ4787(::*rrnB-3*),* □ $^-$, □ *(sucA775), rph-1,* □ *(rhaD-rhaB)568, hsd*R*514, pAB02a*
gltDM	*E. coli* strain BW25113 with *gltD* knocked out, kanamycin resistance removed, and pAB07a	F^-, □ *gltD,* □ *(araD-araB)567,* □ *lacZ4787(::*rrnB-3*),* □ $^-$, □ *(gltD742), rph-1,* □ *(rhaD-rhaB)568, hsd*R*514,* pAB07a
yidAM	*E. coli* strain BW25113 with *yidA* knocked out and kanamycin resistance removed	F^-, □ *ycgG ,* □ *(araD-araB)567,* □ *lacZ4787(::*rrnB-3*),* □ $^-$, □ *(ycgG757), rph-1,* □ *(rhaD-rhaB)568, hsd*R*514, pAB05a*
acrDM	*E. coli* strain BW25113 with *acrD* knocked out, kanamycin resistance removed and pAB08a	F^-, □ *acrD,* □ *(araD-araB)567,* □ *lacZ4787(::*rrnB-3*),* □ $^-$, □ *(acrD790), rph-1,* □ *(rhaD-rhaB)568, hsd*R*514, pAB08a*

B. Plasmids. Cm^R denotes chloramphenicol resistance

Plasmid	Description	Resistance
pAB191	p15A ori; proD promoter driving lacZ	cm^R
pAB01a	p15A ori; proD promoter driving *icdA*$^+$	cm^R
pAB02a	p15A ori; proD promoter driving *sucA*$^+$	cm^R
pAB04a	p15A ori; proD promoter driving *ushA*$^+$	cm^R
pAB05a	p15A ori; proD promoter driving *yidA*$^+$	cm^R
pAB07a	p15A ori; proD promoter driving *gltD*$^+$	cm^R
pAB08a	p15A ori; proD promoter driving *acrD*$^+$	cm^R
pAB09a	p15A ori; proD promoter driving *ompF*$^+$	cm^R

Table S2: Multiple comparison test with Fisher's least significant difference correction for Figures 1 and S1

A. P values for K_S as defined based on maximum density

strain1	strain2	pvals
gltDM	sucAM	0.277439685
gltDM	ompF	0.274988792
gltDM	acrD	0.244454906
gltDM	yidAM	0.159286879
gltDM	icdAM	0.019092548
gltDM	ushAM	0.002393476
gltDM	lacZ	0.001088546
sucAM	ompF	0.995306582
sucAM	acrD	0.934088996
sucAM	yidAM	0.729525705
sucAM	icdAM	0.157771432
sucAM	ushAM	0.02480617
sucAM	lacZ	0.011574528
ompF	acrD	0.938766099
ompF	yidAM	0.733926331
ompF	icdAM	0.159350704
ompF	ushAM	0.025105339
ompF	lacZ	0.011718506
acrD	yidAM	0.792229536
acrD	icdAM	0.181202711
acrD	ushAM	0.029339692
acrD	lacZ	0.013765957
yidAM	icdAM	0.275087137
yidAM	ushAM	0.049525035
yidAM	lacZ	0.02375069
icdAM	ushAM	0.334653207
icdAM	lacZ	0.19013119
ushAM	lacZ	0.713670215

B. P values for maximum growth rate

strain1	strain2	pvals
gltDM	sucAM	0.01939854
gltDM	ompF	0.238885482
gltDM	acrD	0.525854926
tDM	yidAM	0.302429168
gltDM	icdAM	0.006220099
gltDM	ushAM	0.001397867
gltDM	lacZ	0.002582454
sucAM	ompF	0.188045988
sucAM	acrD	0.068919069
sucAM	yidAM	0.144821784
sucAM	icdAM	0.590212968
sucAM	ushAM	0.22666931
sucAM	lacZ	0.348114106
ompF	acrD	0.57334996
ompF	yidAM	0.876562554
ompF	icdAM	0.072240066
ompF	ushAM	0.018104678
ompF	lacZ	0.032461251
acrD	yidAM	0.682166804
acrD	icdAM	0.02369425
acrD	ushAM	0.005491879
acrD	lacZ	0.010086135
yidAM	icdAM	0.053702756
yidAM	ushAM	0.013100694
yidAM	lacZ	0.02369425
icdAM	ushAM	0.489253431
icdAM	lacZ	0.682166804
ushAM	lacZ	0.774996373

Table S3: Multiple comparison test with Fisher's least significant difference correction for Fig. 3F parameters

d:

drug1	drug2	pvals
0	1	0.250997402
0	2	0.077793142
0	3	0.011081724
0	4	0.029323972
1	2	0.535562241
1	3	0.160128315
1	4	0.29875954
2	3	0.431165968
2	4	0.674201812
3	4	0.713483253

k:

drug1	drug2	pvals
0	1	8.48097E-10
0	2	2.71712E-12
0	3	3.43597E-19
0	4	7.51645E-21
1	2	0.329310019
1	3	0.000891365
1	4	0.000125832
2	3	0.017765837
2	4	0.003834185
3	4	0.59466969

K_S

drug1	drug2	pvals
0	1	1.18881E-10
0	2	8.88939E-16
0	3	2.64264E-22
0	4	1.8902E-24
1	2	0.061103401
1	3	8.05512E-05
1	4	4.86639E-06
2	3	0.03425778
2	4	0.00563149
3	4	0.507155071

Table S4: Model parameters and initial conditions

Parameter	Representation	Value	Definition
Maximal growth rate	μ_M	0.5	Maximal growth rate
Half-maximal substrate concentration	K_S	0.004	Concentration to achieve 50% cell growth
Drug sensitivity/ IC_{50}	K	4.2	Concentration of the drug needed to inhibit the cell growth by 50%
Specific death rate	D	0.025	Antibiotic-mediated cell death
Substrate conversion constant	E	5e-7	Efficiency by which substrate is converted into biomass
Substrate carrying capacity	N_m	1e7	Carrying capacity of the environment
Initial substrate concentration	S_0	1	Initial condition for model simulation
Initial cell density	N_0	1e4	Initial condition for model simulation
Coefficient of variance of maximal-growth rate	-	0.05	For calculating the theoretical standard deviation for μ_M
Coefficient of variance of death rate	-	0.2	For calculating the theoretical standard deviation for d
Coefficient of variance of drug sensitivity	-	0.2	For calculating the theoretical standard deviation for k
Coefficient of variance of Half-maximal growth concentration	-	-0.07	For calculating the theoretical standard deviation for K_S

Table S5: Correlation coefficients of metrics of metabolic efficiencies.

Timestamp	S_0	Metric	Correlation between efficiencies from Ks and e
2hr	0.4	N/S	-0.97101
2hr	0.4	Log N/S	-0.97111
6hr	0.4	N/S	-0.97016
6hr	0.4	Log N/S	-0.97040
**Substrate**		**Metric**	**Correlation with *Ks***
Constant at 0.5 at 2hr	0.4	N at constant S	-0.999998
Constant at 0.5 at 6hr	0.4	N at constant S	-0.99995
<0.50	1.0	N at a specific S	-0.998467
<0.04	1.0	N at a specific S	-0.999996

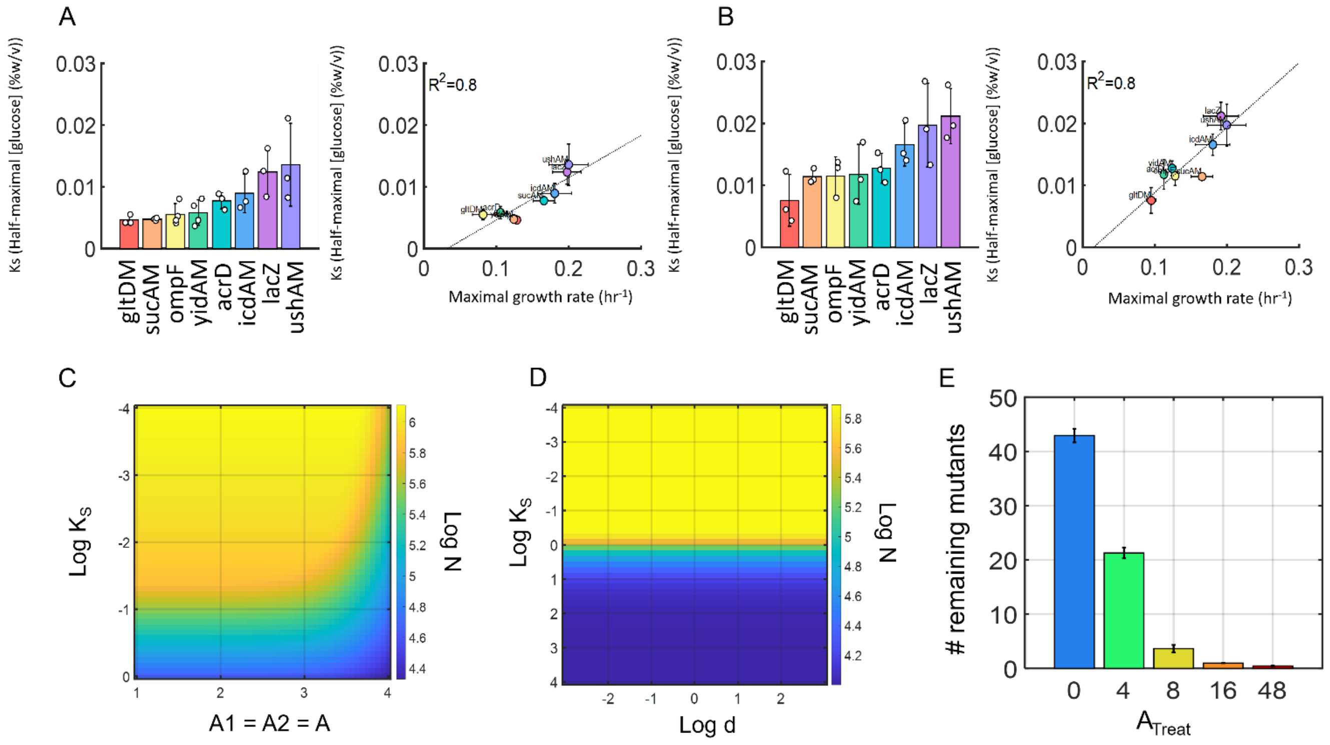

Fig. S1. Quantitative evaluation of K_S and cell density.
This trend was independent of whether biomass or growth rate was used to calculate K_S **(A)**, or whether the maximum density was normalized across strains prior to fitting**(B)**. **(C)** Decreasing the K_S (half-maximal substrate concentration) increases the final cell density, as the cells can grow faster with less amounts of nutrients, dependent on the antibiotic concentration. **(D)** In the absence of antibiotics, the relationship between K_S and cell density is not dependent on the intrinsic death rate. **(E)**For sufficiently high A_{Treat} concentrations where the adaptation limit was exceeded, no mutants are selected for and the entire population is eliminated. In all cases, the error bars represent the standard errors across all 50 iterations.

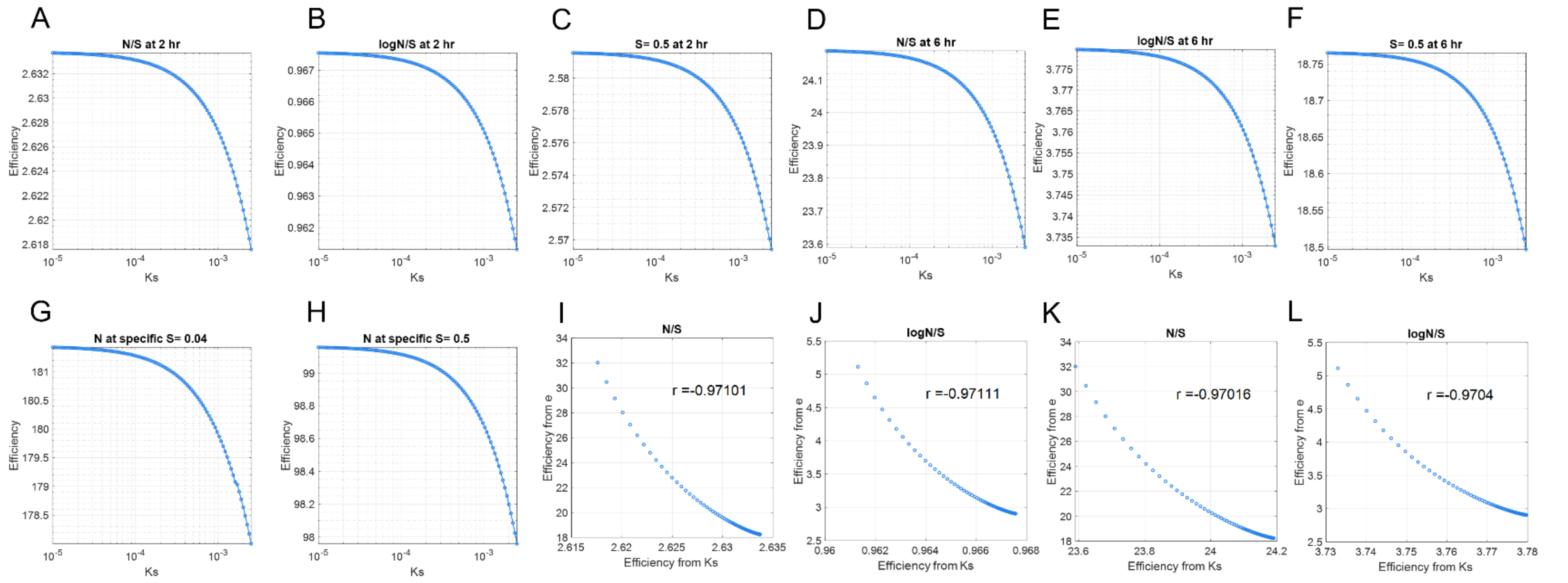

Fig. S2. Metric validation. Four different metrics for metabolic efficiency, generally defined as the amount of biomass generated per unit substrate consumed, are compared as a function of increasing K_S: (1) N/S at 2 **(A)** or 6 **(D)** hours; (2) log N/S at 2 **(B)** or 6 **(E)** hours; (3) N when S is constant and in excess 0.5 at 2 or **(C)** or 6 **(F)** hours; (4): N at a specific S of 0.04 **(G)** or 0.50 **(H)**. Two time points for metrics 1-3 are shown, since this definition depends on time; we note that the trend was independent of the time chosen so long as they fell within exponential growth. In all cases, metabolic efficiency decreases with increasing K_S. Definition 4 is used in the main text. The correlation coefficients for figures**(A)-(H)** are provided in **Table S5.** Two metrics of metabolic efficiency (N/S and log N/S) with variation in K_S and e (substrate conversion constant) at 2hr and 6hr were considered. Since the substrate (S) is fixed, the other two metrics of efficiency (N for a constant S, N for a given S) cannot be calculated. There is a strong negative-linear correlation between K_S and e in all cases. **(I)** Correlation of efficiencies for K_S and e for the metric (N/S) at 2 hr. **(J)** Correlation of efficiencies for K_S and e for the metric (log N/S) at 2 hr. **(K)** Correlation of efficiencies for K_S and e for the metric (N/S) at 6 hr. **(L)** Correlation of efficiencies for K_S and e for the metric (log N/S) at 6 hr.

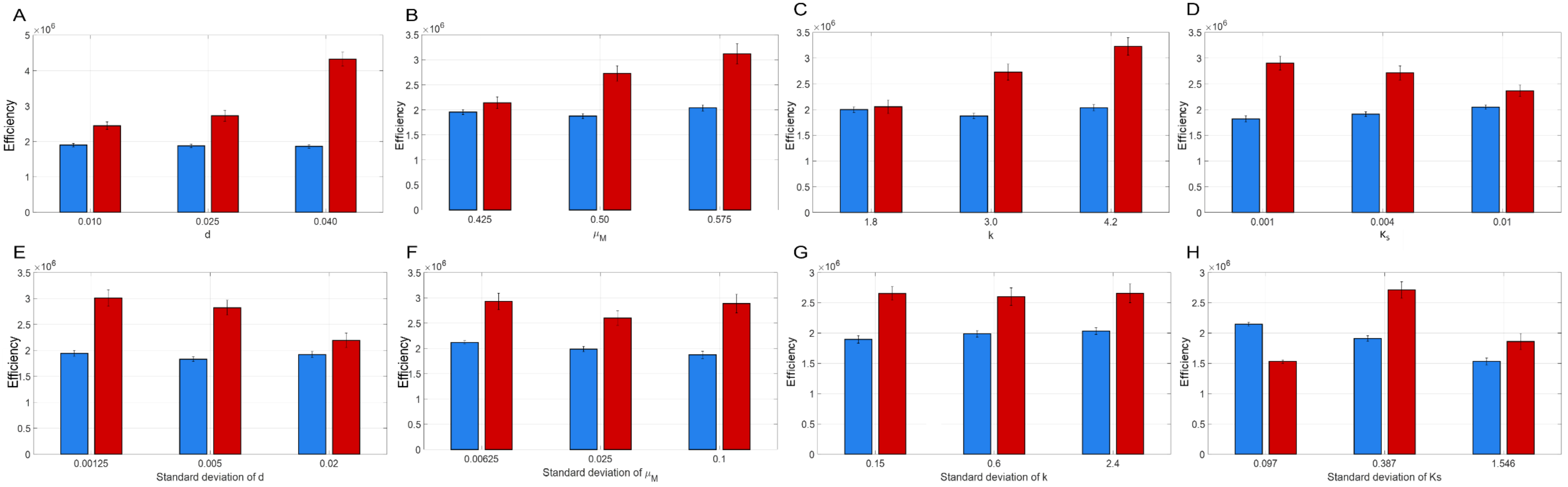

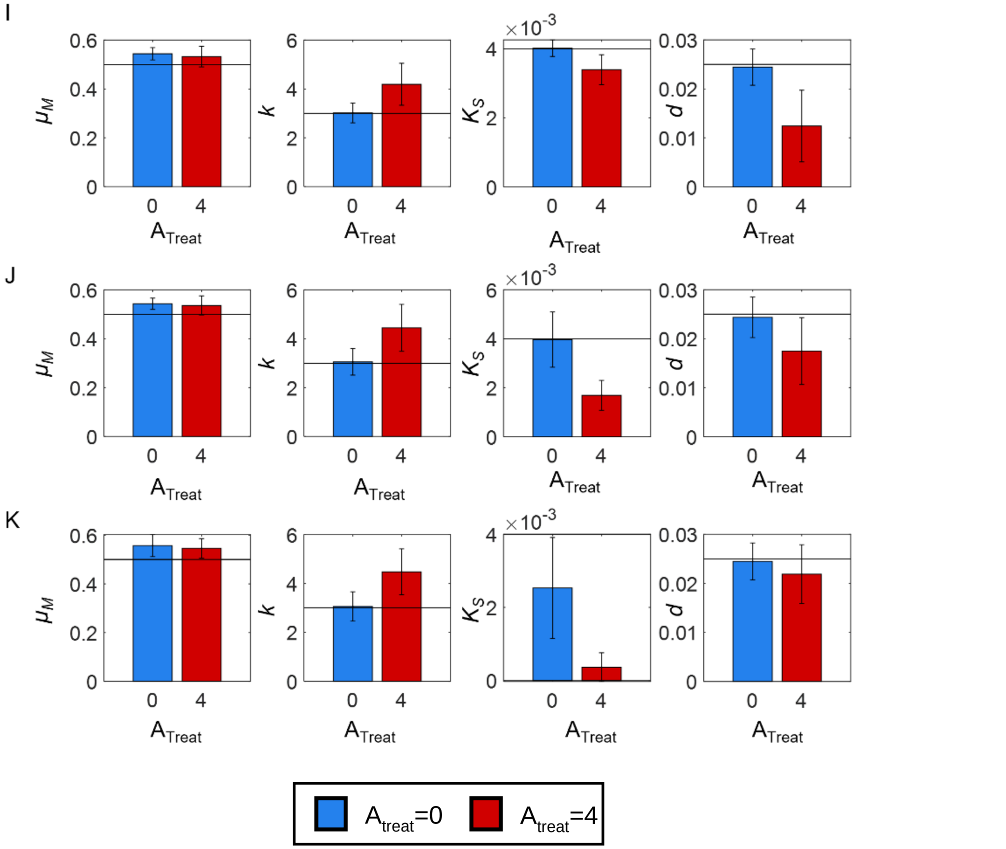

Fig. S3. Parameter sensitivity analysis.
Metabolic efficiency is defined as the cell density at which the substrate concentration drops to 50% of S_0. Each of the four base parameters are considered: **(A)** specific death rate *d,* **(B)** maximal growth rate μ_M, **(C)** drug sensitivity *k,* and **(D)** half-maximal substrate concentration K_S. The parameters *d*, μ_M and K_S, are varied with +/- 3 Standard deviations (SD) from the mean value. The parameter *k* is varied with +/- 2 SDs from the mean to avoid negative values. The mean value for each of the parameters; *d,* μ_M, *k* and K_S; are 0.025, 0.50, 3.0 and 0.004 (the central values in bar plots) respectively. As observed, consistency was achieved with the key result – antibiotics enhance metabolic efficiency at sub-inhibitory drug treatment- through A_{Treat} = 0 (blue) and A_{Treat} = 4 (red) conditions. For all cases, the error bars represent the standard errors across all 50 iterations. Further, the distribution widths are considered for each of the four base parameters, **(E)** specific death rate *d,* **(F)** specific growth rate μ_M, **(G)** drug sensitivity *k,* and **(H)** half-maximal substrate concentration K_S. Metabolic efficiency (y-axis) is defined as the cell density at which the substrate concentration drops to 50% of S_0. For each of the four base parameters, a 3-fold change in their respective standard deviation was considered. Overall, the key result, where antibiotics select for enhanced metabolic efficiency, is robust to changes in the distribution widths/theoretical standard deviations of the parameters. However, results are not obtained when the distribution for K_S is too narrow (ie., 0.97 in D). In all cases, the error bars represent the standard errors across all 50 iterations. Efficiencies are calculated for two evolved conditions, namely where A_{Treat} = 0 (blue) and A_{Treat} = 4 (red). We can observe that K_S, in presence of antibiotic, that is, from A_{Treat} = 0 (blue) to A_{Treat} = 4(red), consistently drops for all the three considered scenarios. This figure established that K_S decreases in the presence of antibiotics. Despite an apparent drop in the efficiency observed in **(Fig. S3H), (Fig. S3I)** shows that there is a consistency in the decrease for K_S. **(I)** Distribution at standard deviation 0.097. **(J)** Distribution at standard deviation 0.387. **(K)** Distribution at standard deviation 1.546.

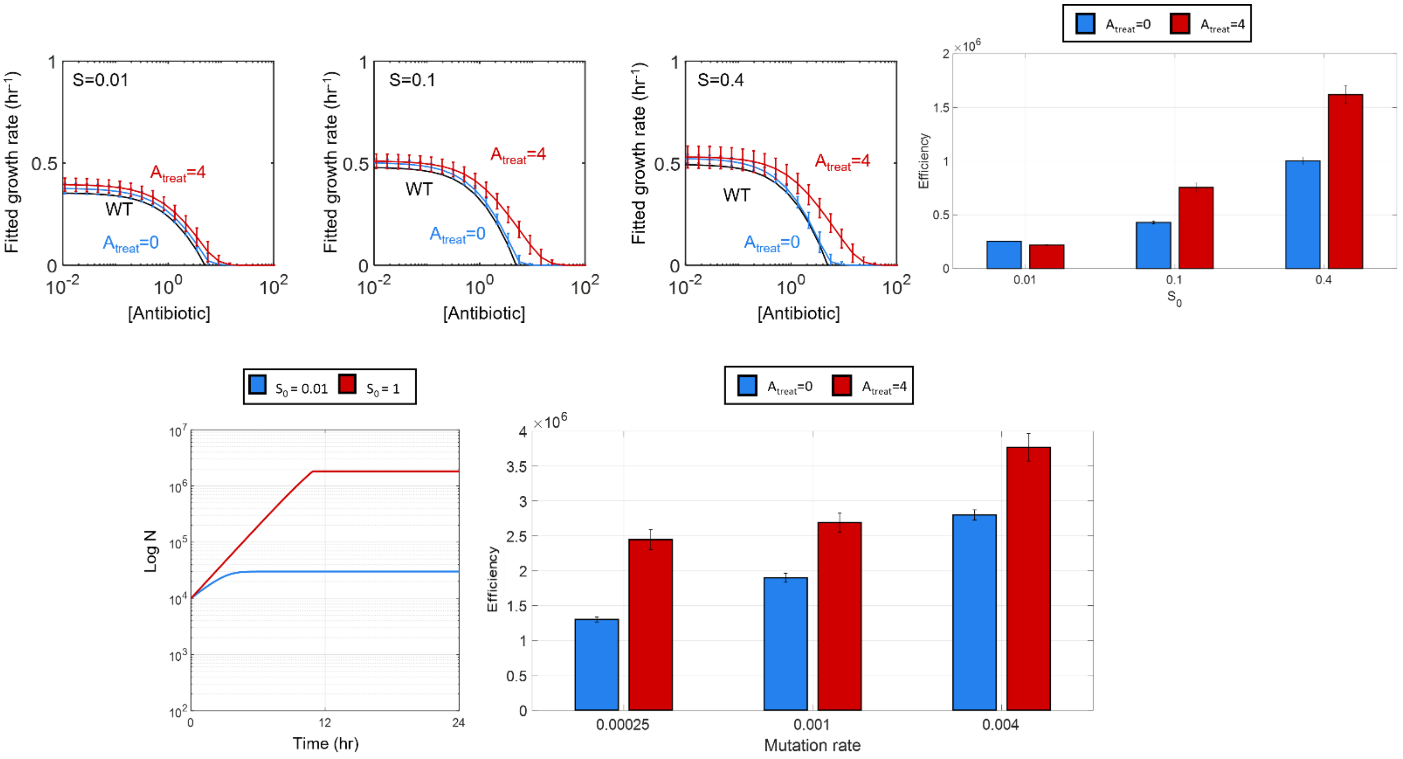

Fig. S4. The effect of initial substrate concentrations (S_0) and mutation rates on metabolic efficiency. Evolution simulations are implemented at various initial substrate concentrations under two different antibiotic treatments (A_{Treat}=0 and A_{Treat}=4), and the evolved populations are characterized. **(A)** IC_{50} curve when S_0 = 0.01; **(B)** IC_{50} curve when S_0 = 0.1; **(C)** IC_{50} curve for S_0 = 0.4. Only B-C shows a shift in drug sensitivity for A_{Treat}=0.4. For **(A)-(C)**, x-axis is antibiotic concentration and y-axis is growth rate. Populations from the last day of evolution are used to estimate the IC_{50} values for A_{Treat}=0 (blue) and A_{Treat}=4 (red). The wild type, WT, (black) is the ancestor population for comparison. **(D)** For relatively high S_0 (0.1 and 0.4), antibiotics enhanced metabolic efficiency consistent with main results. For sufficiently low S_0 (0.01), where the IC_{50} did not shift, no difference in metabolic efficiency was observed. Metabolic efficiency is defined as the cell density at which the substrate concentration drops to 50% of S_0. The error bars represent the standard errors across all the 50 iterations. **(E)** We can observe that at S_0 = 0.01, the cell growth is severely restricted, likely preventing complete evolution over the considered time period. **(F)** A 3-fold change in the mutation rate from its mean (0.001) was considered. Metabolic efficiency is defined as the cell density at which the substrate concentration drops to 50% of S_0. The key result, that antibiotics select enhanced metabolic efficiency at sub-inhibitory drug treatment, is not dependent on the selected mutation rate. In all cases, the error bars represent the standard errors across all 50 iterations. Efficiencies are calculated for two evolved conditions, namely where A_{Treat} = 0 (blue) and A_{Treat} = 4 (red).

Fig. S5 (i). Alternate model 1

(A) Evolved IC_{50} concentrations. Evolution results are investigated for alternative model structure 1 (Equations (5)-(6) in the main text). X-axis is antibiotic concentration and y-axis is growth rate. Populations from the last day of evolution are used to estimate IC_{50} values. IC_{50} values are measured for the drug-free (blue) evolution and for evolved antibiotic treating concentration (A_{Treat}) equal to 4 (red). The wild type, WT, (black) is the ancestor population for comparison. As observed, cells evolved under $A_{Treat}=4$ exhibit a modestly shifted IC_{50} curve.

(B) Antibiotics select for increased metabolic efficiency. As antibiotic treatment concentration increases selected metabolic efficiency also increases. In all cases, error bars represent standard deviation across all 50 iterations.

(C) Evolved parameters. On the last day of evolution, parameter values are collected and weighted by the respective population size. Since substrate utilization *e*, is coupled to K_S, only the latter is shown. Consistent with our main model, K_S decreases with increasing A_{Treat}.

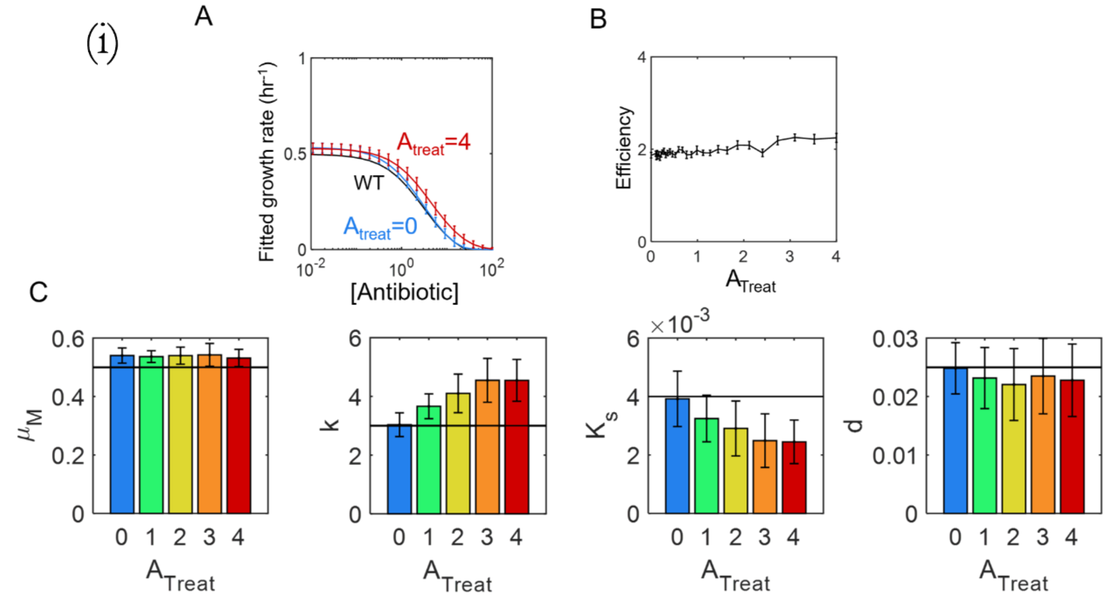

Fig. S5 (ii). Alternate model 2

(A) Evolved IC_{50} concentrations. Evolution results are investigated for alternative model structure 2 (Equations (7)-(8) in the main text). X-axis is antibiotic concentration and y-axis is growth rate. Populations from the last day of evolution are used to estimate IC_{50} values. IC_{50} values are measured for the drug-free (blue) evolution and for evolved antibiotic treating concentration (A_{Treat}) equal to 4 (red). The wild type, WT, (black) is the ancestor population for comparison. As observed, cells evolved under $A_{Treat}=4$ exhibit a modestly shifted IC_{50} curve.

(B) Antibiotics select for increased metabolic efficiency. As antibiotic treatment concentration increases selected metabolic efficiency also increases. In all cases, error bars represent standard deviation across all 50 iterations.

(C) Evolved parameters. On the last day of evolution, parameter values are collected and weighted by the respective population size. Since substrate utilization *e*, is coupled to K_S, only the latter is shown. Consistent with our main model, K_S decreases with increasing A_{Treat}.

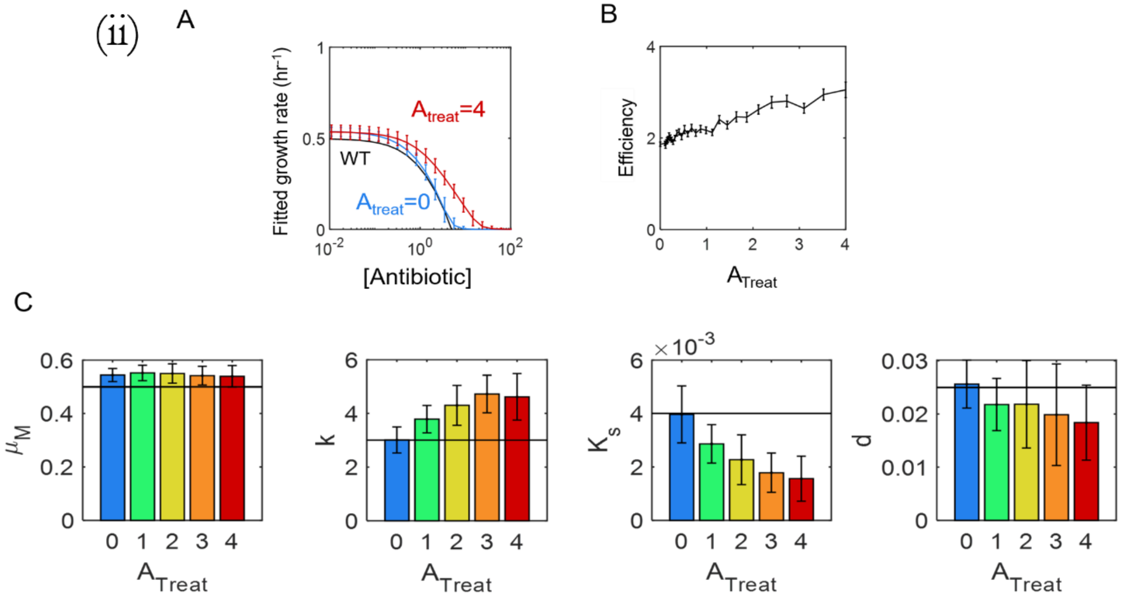

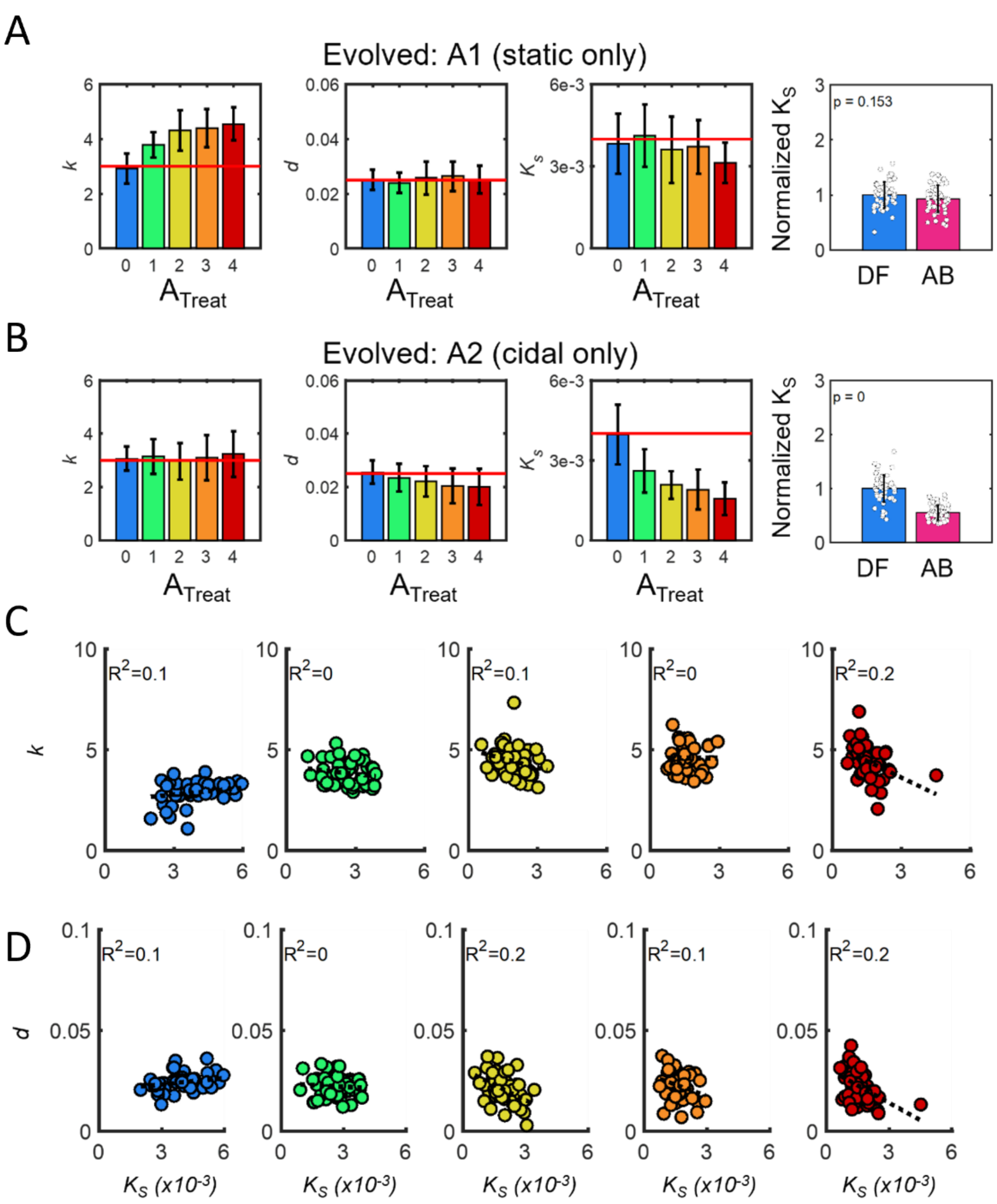

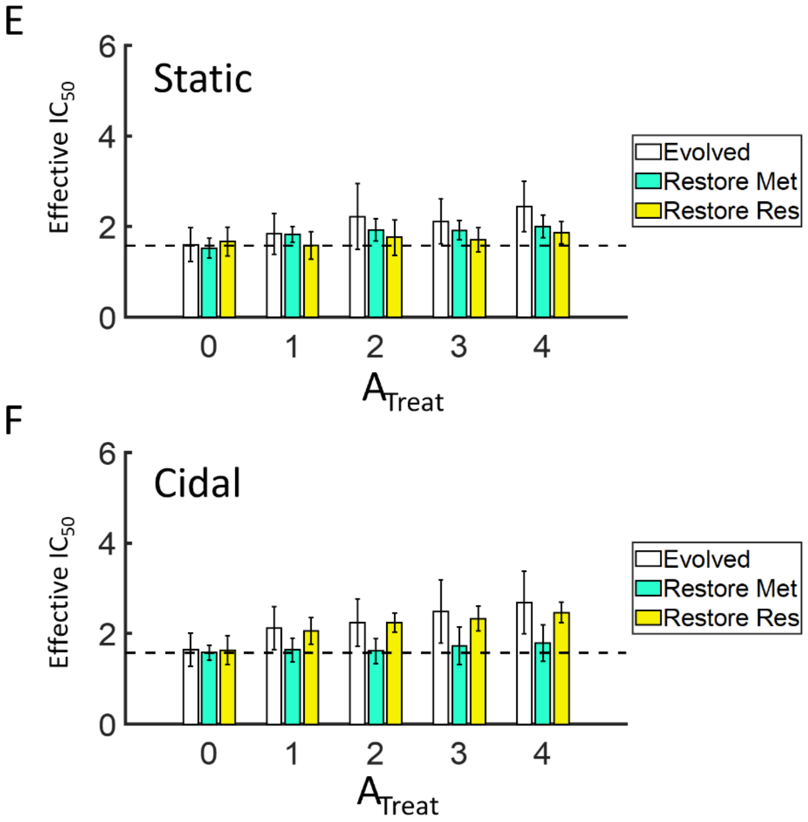

Fig. S6. Evolution subjected to strictly static and strictly cidal activity. (A) For entirely static conditions (A_2=0), only k evolved significantly, with negligible evolutionary changes in K_S. **(B)** For entirely cidal conditions (A_1=0), K_S decreases significantly with an increase A_{Treat} with insignificant changes in k. No, or very weak, correlations between metabolic (K_S) and resistance parameters k **(C)** and d **(D)** within a population under sub-inhibitory antibiotic selection. **(E)** Reversal strategies for a population evolved under a strictly static drug (A_2=0), restoring canonical resistance parameters was sufficient to entirely re-sensitize the evolved population. Comparatively, restoring metabolic resistance parameters had a smaller effect. **(F)** Reversal strategies for a population evolved under a strictly cidal drug (A_1=0), restoring metabolic parameters was sufficient to entirely re-sensitize the evolved population, whereas restoring canonical resistance parameters had minimal effect
